# Extensive Drug-Resistant *Salmonella enterica* Isolated From Poultry and Humans: Prevalence and Molecular Determinants Behind the Co-resistance to Ciprofloxacin and Tigecycline

**DOI:** 10.3389/fmicb.2021.738784

**Published:** 2021-11-25

**Authors:** Norhan K. Abd El-Aziz, Yasmine H. Tartor, Rasha M. A. Gharieb, Ahmed M. Erfan, Eman Khalifa, Mahmoud A. Said, Ahmed M. Ammar, Mohamed Samir

**Affiliations:** ^1^Department of Microbiology, Faculty of Veterinary Medicine, Zagazig University, Zagazig, Egypt; ^2^Department of Zoonoses, Faculty of Veterinary Medicine, Zagazig University, Zagazig, Egypt; ^3^Reference Laboratory for Veterinary Quality Control on Poultry Production, Animal Health Research Institute, Agricultural Research Center, Giza, Egypt; ^4^Department of Microbiology, Faculty of Veterinary Medicine, Matrouh University, Marsa Matruh, Egypt; ^5^Zagazig University Hospital, Zagazig, Egypt

**Keywords:** XDR, *Salmonella enterica*, AcrAB efflux pump, ciprofloxacin, tigecycline

## Abstract

The emergence of extensive drug-resistant (XDR) *Salmonella* in livestock animals especially in poultry represents a serious public health and therapeutic challenge. Despite the wealth of information available on *Salmonella* resistance to various antimicrobials, there have been limited data on the genetic determinants of XDR *Salmonella* exhibiting co-resistance to ciprofloxacin (CIP) and tigecycline (TIG). This study aimed to determine the prevalence and serotype diversity of XDR *Salmonella* in poultry flocks and contact workers and to elucidate the genetic determinants involved in the co-resistance to CIP and TIG. Herein, 115 *Salmonella enterica* isolates of 35 serotypes were identified from sampled poultry (100/1210, 8.26%) and humans (15/375, 4.00%), with the most frequent serotype being *Salmonella* Typhimurium (26.96%). Twenty-nine (25.22%) *Salmonella enterica* isolates exhibited XDR patterns; 25 out of them (86.21%) showed CIP/TIG co-resistance. Exposure of CIP- and TIG-resistant isolates to the carbonyl cyanide 3-chlorophenylhydrazone (CCCP) efflux pump inhibitor resulted in an obvious reduction in their minimum inhibitory concentrations (MICs) values and restored the susceptibility to CIP and TIG in 17.24% (5/29) and 92% (23/25) of the isolates, respectively. Molecular analysis revealed that 89.66% of the isolates contained two to six plasmid-mediated quinolone resistance genes with the predominance of *qepA* gene (89.66%). Mutations in the *gyrA* gene were detected at codon S83 (34.62%) or D87 (30.77%) or both (34.62%) in 89.66% of XDR *Salmonella*. The *tet*(A) and *tet*(X4) genes were detected in 100% and 3.45% of the XDR isolates, respectively. Twelve TIG-resistant XDR *Salmonella* had point mutations at codons 120, 121, and 181 in the *tet(A)* interdomain loop region. All CIP and TIG co-resistant XDR *Salmonella* overexpressed *ramA* gene; 17 (68%) out of them harbored 4-bp deletion in the *ramR* binding region (T-288/A-285). However, four CIP/TIG co-resistant isolates overexpressed the *oqxB* gene. In conclusion, the emergence of XDR *S. enterica* exhibiting CIP/TIG co-resistance in poultry and humans with no previous exposure to TIG warrants an urgent need to reduce the unnecessary antimicrobial use in poultry farms in Egypt.

## Introduction

*Salmonella enterica* subspecies *enterica* serovars are the leading cause of food-borne zoonoses worldwide, accounting for 93.8 million cases of gastroenteritis and 155000 deaths annually (Majowicz et al., 2010). *S. enterica* is common in chickens, turkeys, quails, pheasants, and other game birds and has been isolated at high percentages from commercially reared poultry. Hence, multiple *S. enterica* serovars originating from poultry have been considered the potential source of human salmonellosis through the consumption of contaminated bird meat (Hoelzer et al., 2011; Thomas et al., 2017; Anbazhagan et al., 2019). Furthermore, farms represent a direct hazard to public health (Hoelzer et al., 2011), while indirect infections occur through the contaminated food products from carrier birds harboring *Salmonella* in the vicinity of food production units (Thomas et al., 2017). The spread of multidrug-resistant (MDR, i.e., resistant to at least one agent in three or more antimicrobial categories) *Salmonella* usually stems from the unjustified use of antimicrobials particularly in the poultry industry. Thus, the potential risk to public health posed by transmission of MDR non-typhoidal *Salmonella* (NTS) from poultry (Anbazhagan et al., 2019) warrants the need for an integrative “One Health” approach for NTS surveillance among human, poultry, and animal populations. More alarming than the MDR is the recent emergence of the extensive drug-resistant [XDR, i.e., resistant to one or more agents in all antimicrobial categories except two or fewer from the worksheet for defining and categorizing the isolates according to Magiorakos et al. (2012)] *Salmonella* such as the previously reported *Salmonella* Indiana (Wang et al., 2017) and *Salmonella* Typhi (Klemm et al., 2018; Saeed et al., 2019). There have been scarce data on the prevalence of such threatening XDR isolates in humans and poultry. It is worthy to note that the available studies on the mechanisms of antimicrobial resistance in *S. enterica* and in particular the XDR isolates have focused on the resistance to a single antibiotic (Chen et al., 2017; Zhang et al., 2017) without deciphering the molecular rationale behind the emergence of cross-antibiotic resistance, including to unrelated drugs.

Fluoroquinolone (e.g., ciprofloxacin, CIP) resistance in *Salmonella* species is mainly attributed to chromosomal mutations or the existence of plasmid-mediated quinolone resistance (PMQR) determinants or both (Robicsek et al., 2006). The chromosome-mediated quinolone resistance is commonly arbitrated by two main mechanisms: (1) spontaneous point mutations in the quinolone-resistant determining regions (QRDRs) of topoisomerase subunits, mainly *gyrA* (Hopkins et al., 2005; Aldred et al., 2014; Wasyl et al., 2014) and (2) overexpression of the major efflux pump (AcrAB-TolC) in *Salmonella* efflux systems, which usually leads to reduced cellular drug accumulation (Zhang et al., 2017).

The expression of AcrAB efflux pump is known to be regulated by global regulators (Usui et al., 2013), including the *ramR* gene. *RamR* mutations could increase the expression of *ramA* and *acrAB* genes resulting in the appearance of efflux-mediated MDR phenotype (Abouzeed et al., 2008), including CIP resistance. Because the resistance of *Salmonella* to CIP is often associated with cross-resistance to other antimicrobials (e.g., tetracyclines, beta-lactams, and chloramphenicol) (Hopkins et al., 2005), seeking novel antimicrobials that could inactivate XDR *Salmonella* is becoming a top priority. In this regard, glycylcycline (e.g., tigecycline, TIG) has come out as a promising, novel, and last-resort broad-spectrum agent for managing XDR *Salmonella* infections. However, decreased susceptibility of *S. enterica* to TIG has been observed (Chen et al., 2017). It was hypothesized that TIG resistance may be attributed to three main tetracycline resistance mechanisms (Chopra and Roberts, 2001): (i) mutations within tetracycline-specific efflux proteins, mainly *tet*(A), (ii) mutations in ribosomal protection proteins, *tet*(M), and (iii) enzymatic inactivation, *tet*(X). Additional resistance mechanisms as AcrAB-TolC and OqxAB efflux pumps overexpression may be also involved in such resistance (Tuckman et al., 2000; Hentschke et al., 2010; Chen et al., 2017).

The available information on the resistance mechanisms of *Salmonella* to CIP and TIG suggests a complex mechanistic view that needs to be delineated.

This study was designed to determine the prevalence, serotype diversity, and antimicrobial resistance phenotypes of *S. enterica* isolated from poultry and contact workers. Moreover, we combined molecular approaches with data analyses to investigate the genetic determinants involved in the co-resistance of XDR *Salmonella* isolates to CIP and TIG. Further, we precisely pinpointed the mutational alterations in *ramRA* regulatory gene that lie behind the overexpression of AcrAB-TolC efflux pump, which could confer CIP and TIG co-resistance.

## Materials and Methods

### Sampling

This study was conducted during the period from January 2019 to April 2020. A total of 1210 clinically diseased and recently dead 1-day to 4-week-old poultry including broiler chickens (*n* = 650), ducks (*n* = 150), pigeons (*n* = 160), quails (*n* = 130), and turkeys (*n* = 120) from 25 poultry flocks located in five governorates in Egypt were sampled aseptically. Diseased birds were diarrheic, depressed, and weak with ruffled feathers. Flight inability was observed on pigeons and quails. Gross lesions including enteritis, enlarged liver with necrotic foci, perihepatitis, pericarditis, and unabsorbed yolk sac were observed in recently dead birds. The samples included fresh fecal dropping and cloacal swabs from diarrheic birds as well as liver, spleen, cecum, gall bladder, and yolk sac from recently dead birds. Fluoroquinolones, sulfonamides, penicillins, aminoglycosides, and tetracyclines were administered to the entire flocks for treatment. For prophylaxis, ESB3 (sulfaclozine 30%), lincomycin, oxytetracycline, and tylan premix (tylosin) were frequently used in poultry farms. Moreover, 375 human stool samples were collected from diarrheic poultry workers who were employed in the same flocks from which the poultry samples were obtained. The samples were collected in sterile separate containers and transported immediately in an icebox to the Microbiology Laboratory, Faculty of Veterinary Medicine, Zagazig University, for bacteriological analysis.

### Ethics Approval and Consent to Participate

The study was approved by Zagazig University Institutional Animal Care and Use Committee (ZU-IACUC) (approval number ZU-IACUC/2/F/12/2019). Written informed consent was obtained from the owners for the participation of their animals in this study. The patients/participants provided their written informed consent to participate in this study. Written informed consent was obtained from the individual(s) for the publication of any potentially identifiable data included in this article.

### Isolation and Identification of *Salmonella enterica*

Isolation of *S. enterica* was carried out according to ISO (ISO, 2017). Briefly, 1 g of each sample was suspended in 9 ml of buffered peptone water (BPW, Oxoid, United Kingdom) and incubated at 37°C for 18 ± 2 h. An aliquot of 0.1 ml of the pre-enrichment culture was inoculated into 10 ml of Rappaport Vassiliadis soy broth (RV; Oxoid, United Kingdom) then incubated at 42°C for 24 h. Selective plating was done on xylose lysine deoxycholate agar (XLD, Oxoid, United Kingdom) and salmonella shigella agar (SS; Oxoid, United Kingdom) followed by incubation at 37°C for 24 h. For biotyping, fresh colonies from each pure culture were examined for oxidase, methyl red, Voges–Proskauer, and citrate utilization tests as well as their characteristic reactions on triple sugar iron and lysine decarboxylase agar (Oxoid, United Kingdom) media. Serotyping of *Salmonella* isolates was conducted using commercially available antisera (Denka Seiken Co., Ltd., United Kingdom) according to the antigenic profile (Kauffmann, 1957). Polymerase chain reaction (PCR) of the *invA* gene was performed to confirm *Salmonella* identification (Oliveira et al., 2003). The reference strain *S. enterica* serovar Typhimurium ATCC^®^ 14028^TM^ was used for quality control.

### Antimicrobial Susceptibility Testing

The antimicrobial susceptibilities of all *Salmonella* isolates were evaluated against 24 commercially available antimicrobial agents (Oxoid, Hampshire, England, United Kingdom) using the disk diffusion method (Bauer et al., 1966). The tested antimicrobials were ampicillin (10 μg), amoxycillin-clavulanic acid (20/10 μg), ampicillin-sulbactam (20/10 μg), cefazolin (30 μg), cefuroxime (30 μg), ceftriaxone (30 μg), cefepime (30 μg), cefoxitin (30 μg), ertapenem (10 μg), imipenem (10 μg), meropenem (10 μg), doripenem (10 μg), gentamicin (10 μg), tobramycin (10 μg), amikacin (30 μg), nalidixic acid (30 μg), CIP (5 μg), tetracycline (30 μg), TIG (15 μg), fosfomycin (50 μg), chloramphenicol (30 μg), sulfamethoxazole-trimethoprim (23.75/1.25 μg), aztreonam (30 μg), and colistin (10 μg). The inhibition zone diameters were measured and interpreted according to Clinical and Laboratory Standards Institute (CLSI) and European Committee on Antimicrobial Susceptibility Testing (EUCAST) guidelines (European Committee Antimicrobial Susceptibility Testing (EUCAST), 2018; Clinical Laboratory Standards Institute (CLSI), 2019). The multiple antibiotic resistance (MAR) indices were calculated as described elsewhere (Tambekar et al., 2006). The MDR and XDR *Salmonella* isolates were categorized according to Magiorakos et al. (2012). The analyzed antimicrobials were synchronized with veterinary guidelines (Plumb, 2015). Justification of the selected antimicrobial agents was for monitoring the XDR *Salmonella* isolates (Magiorakos et al., 2012) and with the end goal of public health concern, e.g., TIG, fluoroquinolones, carbapenems, aminoglycosides, and cephalosporins.

To determine the minimum inhibitory concentrations (MIC) of CIP against XDR *Salmonella* isolates, VITEK^®^ 2 (bioMérieux, Marcy L’Étoile, France) testing was performed using AST-GN91 cards (SKU Number: 414780) according to the manufacturer’s instructions. Interpretive correlation of the VITEK^®^ 2 MIC results was applied using the Advanced Expert System (AES^TM^) rules. Moreover, the MICs of TIG and colistin were determined using broth microdilution method, and the interpretive criteria were those reported in the above mentioned CLSI and EUCAST documents. The MIC breakpoints for CIP (≥1 mg/L) and TIG (>2 mg/L) were considered accordingly.

### Phenotypic Detection of the Efflux Pump Activity

The efflux pump activity of XDR *Salmonella* isolates was determined using the ethidium bromide (EtBr) cartwheel method as described previously (Martins et al., 2011). In brief, trypticase soy agar (TSA, Oxoid, United Kingdom) plates containing 0.0 to 2.5 mg/L of EtBr (Sigma-Aldrich, Germany) concentrations were prepared on the same day of the experiment and kept away from light. Each XDR *Salmonella* isolate (approximately 10^8^ CFU/ml) was streaked on an EtBr plate in a cartwheel pattern. The plates were wrapped in aluminum foil and incubated at 37°C overnight. The minimum concentration of EtBr that produced fluorescence of bacterial colonies under Accuris^TM^ E3000 UV Transilluminator (Accuris Instruments, United States) was recorded. A pan-susceptible *Salmonella* Tamale isolate generated during this study was used as a comparative control for fluorescence analysis. The capacity of each XDR *Salmonella* isolate to expel EtBr substrate was graded relative to the control isolate according to the following equation:


$$\text{Efflux}\text{activity}\mathrm{index}=\frac{{\text{MC}}_{\text{EtBr}}\left(\mathrm{XDR}\right)-{\mathrm{MC}}_{\text{EtBr}}\left(\mathrm{REF}\right)}{{\text{MC}}_{\text{EtBr}}\left(\mathrm{REF}\right)}$$

where MC_*EtBr*_ (XDR) represents the minimum EtBr concentration that produces fluorescence of the XDR test isolate. Meanwhile, MC_*EtBr*_ (REF) indicates the minimum EtBr concentration that produces fluorescence of the reference isolate.

The MICs of CIP and TIG were determined by broth microdilution method in the presence of the carbonyl cyanide 3-chlorophenylhydrazone (CCCP, 5 μg/ml; Sigma-Aldrich, United Kingdom) efflux pump inhibitor (EPI), which is known to dissipate the proton-motive force essential for the activity of resistance-nodulation division (RND) family efflux pumps. A significant efflux inhibition activity was considered when a ≥ 4-fold decrease in the MIC values was reported in the presence of EPI (Rushdy et al., 2013; Deng et al., 2014).

### Detection of Plasmid-Mediated Quinolone Resistance Genes

Plasmid DNA was extracted from XDR *Salmonella* isolates using QIAprep Spin Miniprep Kits (Qiagen, Germany) following the manufacturer’s recommendations. PMQR genes, including *qnrA*, *qnrB*, and *qnrS* were amplified through a multiplex PCR assay, while the *qepA*, *aac(6′)-Ib-cr*, *oqxA*, and *oqxB* genes were amplified by uniplex PCRs using previously published oligonucleotide primers (Supplementary Table 1) and cycling conditions (Robicsek et al., 2006; Cattoir et al., 2008; Kim et al., 2009; Lunn et al., 2010). Positive (*S. enterica* serovar Typhimurium ATCC^®^ 14028^TM^) and negative (a reaction mixture without DNA template) controls were included in each run.

### Detection of Tetracycline Resistance Determinants

The plasmid-encoded *tet*(X1 to X5) genes conferring TIG resistance were amplified using multiplex PCR (Ji et al., 2020). The presence of *tet*(A), *tet*(B), and *tet*(M) genes were also examined (Van et al., 2008) using primer sequences depicted in Supplementary Table 1.

### Detection of Mutations in *gyrA*, *tet*(A), and *ramRA* Genes

The QRDR of *gyrA*, *tet*(A), and *ramRA* genes were amplified using PCR followed by DNA sequencing using oligonucleotide primers listed in Supplementary Table 1. Genomic DNA was extracted from overnight cultures of the XDR *Salmonella* isolates using QIAamp DNA Mini kit (Qiagen, Germany) following the manufacturer’s instructions. The PCR amplicons were purified using the QIAquick PCR purification kit (Qiagen, Germany) and sequenced in an ABI 3130 automated DNA Sequencer (Applied Biosystems, United States) using the BigDyeR Terminator v3.1 Cycle Sequencing Kit (Applied Biosystems, United States) following the supplier protocol. Nucleotide sequences were compared with those previously deposited at GenBank using Basic Local Alignment Search Tool (BLAST^1^). Alignment of the nucleotide sequences was performed using the MEGA6 program (Tamura et al., 2013). The amino acid sequences were deduced using the ExPASy (Expert Protein Analysis System) Translate Tool^2^. The respective regions of nucleotide and polypeptide sequences were analyzed for mutational changes by comparison with the complete genome of *S. enterica* serovar Typhimurium LT2 (GenBank accession number NC_003197).

### Quantification of the Transcription Levels of Efflux Pump Genes

Quantitative PCR (qPCR) was used to determine the relative expression levels of *ramA*, *acrB*, and *oqxB* genes using previously published oligonucleotide primers (Supplementary Table 1). Total RNA was extracted from XDR *Salmonella* isolates using QIAamp RNeasy Mini kit (Qiagen, Germany) following the manufacturer’s instructions. The relative quantification was done in triplicates using QuantiTect SYBR Green real-time PCR Kit (Qiagen, Germany) in MX3005P real-time PCR thermal cycler (Agilent, La Jolla, CA, United States) following the manufacturer’s recommendations. Melting curve analysis was conducted to confirm the specificity of the tested assays. The 16S rRNA housekeeping gene was used as a normalizer (Fàbrega et al., 2016), and the fold change values were estimated using 2^–ΔΔCT^ method (Livak and Schmittgen, 2001). A pan-susceptible *Salmonella* Tamale isolate was used as a comparative control.

### Bioinformatics and Data Analyses

Fisher’s exact test was used to determine if there were significant differences between the infection rates with *Salmonella* in the two age groups (i.e., 1–5 days and 1–4 weeks) in bird species. To visualize the overall distribution of the XDR *Salmonella* isolates based on their resistance patterns, a heatmap supported by hierarchical clustering (dendrogram) was generated (Kolde, 2019). To determine the significance of the association between a certain genetic marker and the resistance phenotype in *Salmonella* isolates, Fisher’s exact test and odds ratio (confidence intervals = 95%) were estimated on contingency tables considering the presence of genetic markers as independent variables and the resistance phenotypes as dependent variables. These analyses were done using GraphPad Prism version 8 for Windows, San Diego, CA, United States^3^. To reveal the significance of the genetic markers as classifiers of the isolates as resistant or susceptible to a certain drug, we applied a random forest classification model using an ensemble of 500 trees. The optimal number of random train predictor variables was determined using the *tuneRF* R function at an “out of bag error,” OOB (prediction error) = 0. The mean decrease in the Gini index was used as an indicator of variable importance (the higher the mean decrease in the Gini index, the more important the marker). This analysis was done using R package “Random Forest” (Breiman, 2001) in the R environment (v. 3.6.2). To calculate the correlation, the raw data were converted into binary outcomes, and the correlation significance was determined (*p*-value significance level = 0.05). These analyses were done using R packages *corrplot* and *Hmisc* (Harrell, 2020). Binary distances between isolates of the same host were measured using dist function in R software. Non-metric multidimensional scaling was used to visualize the clustering of isolates belonging to different hosts based on binary distance. This analysis was done using VEGAN package and the function metaMDS in R software^4^.

### Nucleotide Sequence Accession Numbers

The nucleotide sequences of the genes under study were deposited into the GenBank under the following accession numbers: MT725561–MT725589 for *gyrA*, MT725590–MT725614 and MT740093–MT740096 for *tet*(A), and MT743008–MT743036 for *ramRA* genes.

## Results

### Prevalence and Serotypes of *Salmonella* Isolates in Poultry Flocks and Contact Workers

As shown in Table 1, out of 1210 diarrheic and recently dead poultry sampled, 100 (8.26%) were positive for *Salmonella* species. The prevalence of *Salmonella* was 11.54% (75/650), 6.67% (10/150), 3.13% (5/160), 3.85% (5/130), and 4.17% (5/120) in chickens, ducks, pigeons, quails, and turkeys, respectively. Meanwhile, 15 *S. enterica* isolates were isolated from 375 contact worker stool samples (4.00%). The recovered *Salmonella* isolates were confirmed based on conventional phenotypic and molecular identification methods. Statistical analysis revealed that the difference in the infection rates of all birds with *Salmonella* species was significant (*p*-value = 0.04), whereas it was non-significant among each poultry species (*p*-value > 0.05). Typical *Salmonella* colonies were pink with or without black centers on XLD agar, while white colonies with black centers were characteristic on SS agar medium. Biochemical reactions for presumptive identification of *Salmonella* isolates indicated that all tested isolates were positive for methyl red, Simmons’ citrate, and oxidase tests and displayed characteristic reactions on triple sugar iron and lysine decarboxylase agar media, whereas the analyzed isolates were negative for indole and Voges–Proskauer tests. All *Salmonella* isolates were further confirmed by PCR detection of the *invA* gene (fragment size = 284 bp).

**TABLE 1:** *Salmonella* prevalence and XDR isolates recovered from clinically diseased, recently dead birds, and contact poultry workers.

**Host**	**Examined no.**	**Infected no. (prevalence %)**	**1–5 days old**			**1–4 weeks old**			***p*-value^e^**
			**Examined no.**	***Salmonella*- positive (%)^a^**	**XDR isolates (%)^b^**	**Examined no.**	***Salmonella*- positive (%)^a^**	**XDR isolates (%)^b^**	
Poultry	Broiler chicken (650)	75 (11.54)	280	40 (14.29)	8 (20.00)	370	35 (9.46)	9 (25.71)	0.06
	Duck (150)	10 (6.67)	40	5 (12.50)	0 (0.00)	110	5 (4.55)	3 (60.00)	0.13
	Pigeon (160)	5 (3.13)	65	3 (4.62)	1 (33.33)	95	2 (2.11)	0 (0.00)	0.39
	Quail (130)	5 (3.85)	50	3 (6.00)	2 (66.67)	80	2 (2.50)	1 (50.00)	0.37
	Turkey (120)	5 (4.17)	55	4 (7.27)	0 (0.00)	65	1 (1.54)	0 (0.00)	0.17
	Total (1,210)	100 (8.26)	490	55 (11.22)	11(20.00)	720	45 (6.25)	13 (28.89)	0.04*
Poultry workers	375	15 (4.00)	5 XDR (1.3%^c^, 33.3%^d^)						

*XDR, extensive drug resistant.*

*^*a*^The percentages were calculated according to the total number of examined birds in each poultry species.*

*^*b*^The percentages were calculated according to the total number of recovered *Salmonella* isolates from each poultry species.*

*^*c*^The percentage was calculated according to the total number of examined poultry workers.*

*^*d*^The percentage was calculated according to the total number of infected poultry workers.*

*^*e*^*p*-values obtained by Fisher’s exact test refer to the differences between young (1–5 days) and old (1–4 weeks) birds infected with *Salmonella*.*

Asterisk (*) indicates a significant value.

In all, 35 *Salmonella* serotypes were identified among the 115 isolates originating from poultry and humans using a classical agglutination assay. Regardless of the isolate source, the most frequent serotype was *Salmonella* Typhimurium (26.96%) followed by *Salmonella* Enteritidis (11.30%), *Salmonella* Infantis (6.96%), *Salmonella* Kentucky (5.22%), and *Salmonella* Newport (3.48%); other *Salmonella* serotypes were reported by lower frequencies (Supplementary Table 2). The distribution of *Salmonella* serotypes varied within host species. Typhoidal *Salmonella* serotypes Typhi and Paratyphi C were detected among the human isolates only (3/15; 20% each), whereas all the subspecies *enterica* of poultry origin were categorized as NTS. It was noted that *Salmonella* Typhimurium was the predominant serotype in chickens (22/75; 29.33%) followed by *Salmonella* Enteritidis (12/75; 16.00%). However, certain *Salmonella* serovars were reported and characterized only for ducks (*Salmonella* Derby and *Salmonella* Larochelle), pigeons (*Salmonella* Alfort and *Salmonella* Wingrove), turkeys (*Salmonella* Vejle and *Salmonella* Apeyeme), or quails (*Salmonella* Shangani and *Salmonella* Jedburgh).

### Antimicrobial Susceptibilities and Selection of XDR Isolates

As depicted in Supplementary Table 3, the highest level of resistance was recorded for amoxycillin-clavulanic acid (99.13%) followed by cefazolin (94.78%), nalidixic acid (77.39%), cefoxitin (76.52%), tetracycline (74.78%), and cefepime (73.04%). More than 50% of the isolates exhibited resistance to each of tobramycin, cefuroxime, chloramphenicol, ceftriaxone, sulfamethoxazole-trimethoprim, and ampicillin. CIP and TIG resistance were observed in 51.30 and 24.35% of *S. enterica* isolates, respectively. However, the carbapenems including ertapenem, imipenem, meropenem, and doripenem displayed the maximum activity against the isolates (70.43, 79.13, 89.57, and 93.04%, respectively). Only one isolate of chicken origin (*Salmonella* Tamale; 0.87%), was pan-susceptible to the tested antimicrobials. According to the antimicrobial resistance profile, 73.9% (*n* = 85) of the isolates were MDR (MAR index = 0.13–0.71) and 25.22% (*n* = 29) were XDR (MAR index = 0.63–0.88), of which 24 originated from poultry and only 5 were of human origin (Table 1). *Salmonella* Typhimurium accounts for almost half of the XDR isolates (12/29; 41.38%), of which 8 (66.67%) were human isolates and 4 (33.33%) were of chicken origin.

**FIGURE 1:**
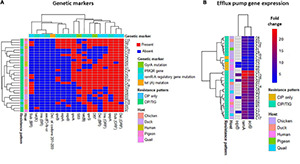
Heatmap supported by hierarchical clustering (dendrogram) depicting the overall distribution of genetic markers **(A)** and efflux pumps gene expression **(B)** in the 29 XDR *Salmonella* isolates. The isolates are color-annotated based on their hosts (C, chicken; D, duck; H, human; P, pigeon, and Q, quails) and resistance pattern [i.e., to CIP, ciprofloxacin (*n* = 4) and to both CIP and TIG (*n* = 25)]. The genetic markers are color-annotated based on their type. BR, binding region; ORF, open reading frame.

As shown in Table 2 and Supplementary Table 4, all XDR *Salmonella* isolates (*n* = 29) were CIP-resistant (MIC = 2–128 μg/ml), and 25 (86.21%) were TIG-resistant (MIC = 4–32 μg/ml). It is worthy of note that four (13.79%) XDR isolates were uniquely resistant to CIP (MIC = 4–8 μg/ml), while 25 isolates (86.21%) showed CIP/TIG co-resistance (Figure 1).

**TABLE 2:** Phenotypic characterization of XDR *Salmonella* isolates recovered from poultry and human origins.

**Isolate no.**	**Isolate code**	**Serovar**	**Source**	**Antimicrobial resistance pattern^a^**	**MAR index**	**MIC (μg/ml)^b^**		**Efflux Pump activity**		**MIC (μg/ml)**	
						**CIP**	**TIG**	**MC_EtBr_ (μg/ml)**	**Index^c^**	**CIP + CCCP**	**TIG + CCCP**
1	H1	Typhimurium	Human stool	AM, AMC, SAM, CZ, CXM, CRO, FEB, FOX, IPM, TOB, NA, CIP, TE, TIG, FOS, C, SXT, ATM, CT	0.79	16	4	1	3	4	0.5
2	C1	Typhimurium	Chicken muscle	AMC, SAM, CZ, CXM, CRO, FEB, FOX, CN, TOB, NA, CIP, TE, TIG, FOS, C, SXT, ATM, CT	0.75	32	4	1	3	16	1
3	D1	Untypable	Duck cloacal swab	AM, AMC, SAM, CZ, CXM, CRO, FEB, FOX, IPM, TOB, NA, CIP, TE, TIG, FOS, C, SXT, CT	0.75	64	16	2	7	16	2
4	C2	Magherafelt	Chicken liver	AM, AMC, SAM, CZ, CXM, CRO, FEB, FOX, TOB, NA, CIP, TE, TIG, FOS, C, SXT, CT	0.71	16	4	1	3	2	0.5
5	C3	Typhimurium	Chicken spleen	AM, AMC, SAM, CZ, CXM, CRO, FEB, FOX, CN, TOB, NA, CIP, TE, FOS, C, ATM, CT	0.71	4	2	0.5	1	0.5	2
6	C4	Takoradi	Chicken cecum	AM, AMC, SAM, CZ, CXM, CRO, FEB, FOX, CN, TOB, NA, CIP, TE, TIG, FOS, C, SXT, CT	0.75	32	8	1.5	5	8	2
7	C5	Labadi	Chicken gall bladder	AM, AMC, SAM, CZ, CXM, CRO, FEB, FOX, CN, TOB, NA, CIP, TE, TIG, FOS, C, SXT, ATM	0.75	64	16	2.5	9	8	1
8	C6	Typhimurium	Chicken liver	AM, AMC, SAM, CZ, CXM, CRO, FEB, FOX, CN, AK, NA, CIP, TE, TIG, FOS, C, SXT, ATM	0.75	2	4	1	3	0.5	2
9	Q1	Jedburgh	Quail dropping	AM, AMC, SAM, CZ, CXM, CRO, FEB, FOX, TOB, NA, CIP, TE, TIG, FOS, C, SXT, ATM, CT	0.75	64	8	1.5	5	8	1
10	Q2	Alfort	Quail liver	AM, AMC, SAM, CZ, FEB, FOX, IPM, TOB, NA, CIP, TE, TIG, FOS, C, SXT, ATM, CT	0.71	64	16	2	7	16	2
11	C7	Typhimurium	Chicken muscle	AM, AMC, CZ, CXM, CRO, FEB, FOX, CN, TOB, NA, CIP, TE, TIG, FOS, C, SXT, ATM, CT	0.75	32	8	1.5	5	4	1
12	C8	Typhimurium	Chicken spleen	AM, AMC, CZ, CXM, FEB, FOX, IPM, TOB, NA, CIP, TE, TIG, FOS, SXT, ATM, CT	0.67	16	16	2	7	8	2
13	D2	Untypable	Duck muscle	AM, AMC, SAM, CZ, FEB, FOX, IPM, ETP, NA, CIP, TE, TIG, FOS, C, SXT, ATM, CT	0.71	8	4	1	3	2	1
14	C9	Blegdam	Chicken muscle	AMC, CZ, CXM, CRO, FEB, FOX, CN, NA, CIP, TE, TIG, FOS, C, SXT, ATM, CT	0.67	2	4	0.5	1	0.25	0.5
15	C10	Infantis	Chicken liver	AMC, CZ, CXM, CRO, FOX, IPM, CN, TOB, NA, CIP, TE, TIG, FOS, C, SXT, CT	0.67	2	4	0.5	1	2	2
16	C11	Enteritidis	Chicken egg yolk	AMC, CZ, CXM, CRO, FEB, FOX, AK, NA, CIP, TE, TIG, FOS, C, SXT, ATM, CT	0.67	2	4	0.5	1	0.5	1
17	H2	Typhimurium	Human stool	AM, AMC, CZ, CXM, CRO, FEB, FOX, AK, NA, CIP, TIG, FOS, C, SXT, ATM, CT	0.67	16	8	1.5	5	4	2
18	H3	Paratyphi C	Human stool	AM, AMC, SAM, CZ, CXM, CRO, FEB, FOX, CN, TOB, AK, NA, CIP, TE, TIG, FOS, C, SXT, ATM, CT	0.83	2	4	0.5	1	2	4
19	H4	Typhimurium	Human stool	AM, AMC, CZ, CXM, CRO, FEB, FOX, CN, TOB, AK, NA, CIP, TE, FOS, C, SXT, ATM, CT	0.75	4	0.5	0.5	1	2	0.5
20	C12	Typhimurium	Chicken muscle	AMC, SAM, CZ, FEB, FOX, CN, TOB, NA, CIP, TE, TIG, FOS, C, SXT, ATM, CT	0.67	2	4	1	3	0.5	2
21	C13	Bardo	Chicken liver	AM, AMC, CZ, CXM, CRO, FEB, FOX, IPM, ETP, MEM, DOR, AK, NA, CIP, TE, TIG, FOS, C, SXT, ATM, CT	0.88	128	32	2.5	9	32	2
22	C14	Sandiego	Chicken cecum	AM, AMC, SAM, CZ, CXM, CRO, FEB, FOX, CN, TOB, NA, CIP, TE, TIG, FOS, C, SXT, CT	0.75	32	16	2	7	8	2
23	C15	Typhimurium	Chicken liver	AM, AMC, CZ, CXM, CRO, FEB, FOX, CN, AK, NA, CIP, TE, FOS, C, SXT, ATM, CT	0.71	4	0.5	0.5	1	4	0.5
24	C16	Magherafelt	Chicken cecum	AM, AMC, SAM, CZ, CXM, CRO, FEB, FOX, IPM, ETP, MEM, DOR, TOB, NA, CIP, TE, TIG, FOS, C, SXT, CT	0.88	32	16	2	7	4	4
25	Q3	Jedburgh	Quail liver	AM, AMC, CZ, CXM, CRO, FEB, FOX, CN, TOB, NA, CIP, TE, TIG, FOS, SXT, ATM, CT	0.71	64	16	2	7	32	2
26	P1	Wingrove	Pigeon liver	AM, AMC, SAM, CZ, CXM, CRO, FEB, FOX, TOB, NA, CIP, TE, TIG, FOS, C, SXT, ATM	0.71	2	4	0.5	1	0.5	4
27	D3	Untypable	Duck muscle	AMC, SAM, CZ, CXM, FEB, FOX, CN, TOB, NA, CIP, TE, TIG, FOS, C, SXT, CT	0.67	2	4	1	3	2	2
28	H5	Typhimurium	Human stool	AM, AMC, SAM, CZ, CRO, FOX, IPM, ETP, MEM, DOR, CN, TOB, NA, CIP, TE, FOS, C, SXT, CT	0.79	8	1	0.5	1	4	1
29	C17	Typhimurium	Chicken muscle	AMC, SAM, CZ, CRO, FOX, CN, TOB, NA, CIP, TE, TIG, FOS, C, SXT, CT	0.63	32	8	1.5	5	16	4

*XDR, extensive drug resistant; MAR, multiple antibiotic resistance; MIC, minimum inhibitory concentration; MC_*EtBr*_, minimum ethidium bromide concentration; AM, ampicillin; AMC, amoxycillin-clavulanic acid; SAM, ampicillin-sulbactam; CZ, cefazolin; CXM, cefuroxime; CRO, ceftriaxone; FEB, cefepime; FOX, cefoxitin; IPM, imipenem; ETP, ertapenem; MEM, meropenem; DOR, doripenem; CN, gentamicin; TOB, tobramycin; AK, amikacin; NA, nalidixic acid; CIP, ciprofloxacin; TE, tetracycline; TIG, tigecycline; FOS, fosfomycin; C, chloramphenicol; SXT, sulfamethoxazole-trimethoprim; ATM, aztreonam; CT, colistin; CCCP, carbonyl cyanide 3-chlorophenylhydrazone; H, human; C, chicken; D, duck; Q, quail; P, pigeon; CLSI, Clinical and Laboratory Standards Institute.*

*^*a*^Susceptibility of *Salmonella* isolates to colistin (CT) was determined using the broth microdilution method. Colistin MIC range was 4–32 μg/ml for resistant *Salmonella* isolates, while it was 0.5–2 μg/ml for those that were colistin sensitive (Nos. 7, 8, and 26).*

*^*b*^MICs for CIP and TIG were interpreted according to the relevant CLSI document.*

*^*c*^The efflux pump index provided the range of efflux activity, which was reported for each strain in comparison with a pan-susceptible *Salmonella* Tamale control isolate (assigned a value of 0.25 μg/ml).*

### Phenotypic Detection of the Efflux Pump Activity

The efflux activity of XDR *Salmonella* isolates was assessed by testing the ability of the bacteria to pump EtBr out of the cell using the cartwheel test. Fluorescence of *Salmonella* isolates which grew as a confluent mass along a radial line of TSA plates containing increasing concentrations of EtBr was reported. The minimum concentration of EtBr and the efflux activity index for each *Salmonella* isolate are illustrated in Table 2. The fluorescence of tested *Salmonella* was lower than that produced by the control (pan-susceptible *Salmonella* Tamale), which fluoresced at 0.25 μg/ml EtBr. Two chicken isolates (2/29; 6.90%) began to fluoresce at an EtBr concentration of 2.5 μg/ml, whereas 6 isolates from various poultry types fluoresced at 2 μg/ml EtBr. The remaining *Salmonella* isolates (*n* = 21) fluoresced at EtBr concentration range of 0.5–1.5 μg/ml. As shown in Table 2, the MIC values of CIP were reduced by one–threefold in the presence of CCCP in 25 out of 29 (86.21%) CIP-resistant isolates; among them, only 5 isolates (17.24%) showed reverse CIP resistance patterns (MIC = 0.5 μg/ml), while one–fourfold decrease in the TIG MICs was reported in 23 out of 25 (92%) TIG-resistant isolates. Based on these results, we continued the study by further molecular evaluation of the contribution of different mechanisms to the resistance to CIP and TIG, known to be substrates for the efflux pump system, in the 29 XDR and EtBr cartwheel positive *Salmonella* clinical isolates.

### Contribution of Plasmid-Mediated Quinolone Resistance Determinants and *gyrA* Mutations in Fluoroquinolone Resistance

The existence of PMQR genes, *qnrA*, *qnrB*, *qnrS*, *qepA*, *oqxA*, *oqxB*, and *aac(6′)-lb-cr*, was examined in XDR *Salmonella* using conventional PCRs. Data analyses revealed that three XDR *Salmonella* isolates (10.34%) did not have any of the studied PMQR genes, while none of the isolates harbored all the PMQR genes. The majority of the isolates (89.66%) contained two to six PMQR genes. The *qepA* gene was the most frequent PMQR determinant (89.66%) followed by *qnrS* (62.07%), *qnrA* (55.17%), and *qnrB* (27.59%). Six isolates (20.69%) were tested positive for both *oqxA* and *oqxB* genes, whereas one isolate (3.45%) was tested positive for each of *oqxA* and *oqxB* genes. The *aac(6′)-lb-cr* was not detected in any of the tested *Salmonella* isolates (Table 3).

**TABLE 3:** Existence of plasmid-mediated quinolone resistance genes, *tet* genes, mutations in the target [*gyrA*, *tet*(A)] and regulatory (*RamR-A*) genes, and expression of efflux pump genes (*ramA* and *acrB*) in XDR *Salmonella enterica* serovars showing ciprofloxacin and/or tigecycline resistance.

**Isolate no.**	**Serovar**	**PMQR pattern**	***tet* genes**	**Target gene mutation^a^**		***RamR-A* regulatory gene mutation^a^**		**Expression level**			**Accession number^b^**
				***gyrA***	***tet*(A)**	***ramR*/ramR**	***ramR* binding site**	***ramA***	***acrB***	***oqxB***	
1	Typhimurium	*qnrA*; *qepA*	*tet*(A)	S83A; D87V	–	F146L; Del G_69_–A_72_; Ins (3 nt) C_235_	T-97C; Del T_–288_/A_–285_	10.04	7.61	ND	MT725561; MT725590; MT743008
2	Typhimurium	*qnrA*; *qnrS*; *qepA*	*tet*(A); *tet*(B)	S83Y; D87R	L121P	F146L; Ins (3 nt) G_484_; Ins (3 nt) C_235_; Del A_67_–G_69_	T-97C; Del T_–288_/A_–285_	10.10	8.09	ND	MT725562; MT725591; MT743009
3	Untypable	*qnrA*; *qnrB*; *qnrS*; *qepA*	*tet*(A); *tet*(B)	S83T; D87R	L121T	G186R; F149L; D104G; N87S; H82R; Ins (7 nt) G_321_; Ins (5 nt) C_252_; Del T_65_–G_69_	T-97C; V28M; Del T_–288_/A_–285_	16.33	10.84	ND	MT725563; MT725592; MT743010
4	Magherafelt	*qnrS*; *qepA*	*tet*(A)	S83L	–	F148L; Ins (7 nt) G_319_; Ins (3 nt) C_235_; Del G_69_–A_72_	T-97C; Del T_–288_/A_–285_	10.55	9.92	ND	MT725564; MT725593; MT743011
5	Typhimurium	*qnrA*; *qepA*; *oqxA*; *oqxB*	*tet*(A)	S83F	L121P	F148L; Ins (6 nt) T_319_; Ins (3 nt) C_235_; Del G_69_–A_72_	T-97C	5.10	2.83	1.4	MT725565; MT725594; MT743012
6	Takoradi	*qnrS*; *qepA*; *oqxA*; *oqxB*	*tet*(A); *tet*(B)	S83F; D87Y	F120S; L121A	G186R; F149L; D104G; N87S; H82R; Ins (7 nt) G_321_; Ins (5 nt) C_252_; Del T_65_–G_68_	V28M; Del T_–288_/C_–285_	12.21	11.12	4.3	MT725566; MT725595; MT743013
7	Labadi	*qnrA*; *qnrS*; *qepA*	*tet*(A); *tet*(B)	S83V; D87S	L121P	F149L; A102V; N87S; H82R; Ins (7 nt) G_324_; Ins (5 nt) C_252_; Del T_65_–G_69_	I40L; V28M; Del T_–288_/A_–285_; Del G_–52_/A_–49_	22.68	20.90	ND	MT725567; MT725596; MT743014
8	Typhimurium	*qnrS*; *qepA*	*tet*(A); *tet*(B)	S83Y	–	G182R; F145L; G109S; L72F; Del G_307_–C_309_; Del G_251_–C_254_; Del G_64_–G_69_	T-97C; I40L	8.11	6.62	ND	MT725568; MT725597; MT743015
9	Jedburgh	*qnrA*; *qepA*	*tet*(A)	S83L; D87N	–	F145L; S84G; G182R; Del C_307_–G_309_; Del C_254_–A_257_; Del T_65_–G_69_	T-97C; Del T_–288/_A_–285_; Del T_–89_/T_–87_	11.35	8.12	ND	MT725569; MT725598; MT743016
10	Alfort	*qnrA*; *qnrB*; *qnrS*; *qepA*; *oqxA*; *oqxB*	*tet*(A); *tet*(B)	D87F	G181D	G186R; F149L; D104G; N87S; H82R; Ins (7 nt) G_321_; Ins (5 nt) C_252_; Del T_65_–G_69_	V28M; Del T_–288/_A_–285_	15.86	13.66	10.1	MT725570; MT725599; MT743017
11	Typhimurium	*qnrA*; *qnrB*; *qnrS*; *qepA*; *oqxA*; *oqxB*	*tet*(A)	S83F; D87A	G181D	G186R; F149L; D104G; N87S; H82R; Ins (7 nt) G_321_; Ins (5 nt) C_252_; Del T_65_–G_69_	T-97C; V28M; Del T_–288/_A_–285_	12.80	8.24	5.4	MT725571; MT725600; MT743018
12	Typhimurium	*qnrS*; *qepA*	*tet*(A); *tet*(B)	S83E	–	G182R; F145L; G109S; L72F; Del G_251_–C_254_; Del T_65_–G_69_; Del G_307_–C_309_	I40L; Del T_–288/_A_–285_; Del T_–89_/T_–87_	14.83	9.51	ND	MT725572; MT740093; MT743019
13	Untypable	–	*tet*(A); *tet*(B)	S83F	–	F148L; Ins (7 nt) G_319_; Ins (3 nt) C_235_; Del G_69_–A_72_	T-97C; Del T_–288/_A_–285_	10.91	7.44	ND	MT725573; MT725601; MT743020
14	Blegdam	*qnrA*; *qepA*	*tet*(A); *tet*(B)	S83F	–	F149L; Ins (7 nt) G_321_; Ins (5 nt) C_252_; Del T_65_–G_69_	T-97C	7.11	4.38	ND	MT725574; MT725602; MT743021
15	Infantis	*qnrA*; *qepA*	*tet*(A)	–	L121P	F145L; Del G_69_–A_72_; Del G_251_–C_254_; Del G_307_–C_309_	T-97C	7.31	4.68	ND	MT725575; MT725603; MT743022
16	Enteritidis	*qnrA*; *qepA*	*tet*(A); *tet*(B)	–	–	F145L; Del G_69_–A_72_; Del G_251_–C_254_; Del G_307_–C_309_	T-97C	7.61	5.20	ND	MT725576; MT725604; MT743023
17	Typhimurium	*qnrA*; *qnrS*; *qepA*	*tet*(A); *tet*(B)	S83F	–	Ins (6 nt) C_411_; Ins (3 nt) G_377_; Del G_307_–G_309_; Del G_251_–C_254_; Del G_69_–A_72_	Del T_–288/_A_–285_	12.19	10.73	ND	MT725577; MT725605; MT743024
18	Paratyphi C	*qnrS*; *qepA*	*tet*(A); *tet*(X4)	D87Y	–	F148L; Ins (7 nt) G_319_; Ins (3 nt) C_235_; Del G_69_–A_72_	–	6.21	2.50	ND	MT725578; MT725606; MT743025
19	Typhimurium	*qnrA*; *qnrB*; *qnrS*; *qepA*; *oqxA*; *oqxB*	*tet*(A)	S83V	–	F145L; Del G_307_–C_309_; Del C_254_–A_257_; Del G_69_–A_72_	T-97C	4.60	3.97	1.1	MT725579; MT725607; MT743026
20	Typhimurium	*qnrB*; *qnrS*; *qepA*	*tet*(A); *tet*(B)	–	–	F150L; N88S; H83R; Ins (7 nt) G_326_; Ins (5 nt) C_257_; Ins (5 nt) G_64_	–	8.32	3.84	ND	MT725580; MT725608; MT743027
21	Bardo	*qnrA*; *qnrB*; *qnrS*; *qepA*; *oqxA*; *oqxB*	*tet*(A)	D87Y	–	F147L; Ins (4 nt) A_319_; Ins (3 nt) C_235_; Del G_69_–A_72_	T-97C; Del T_–288_/A_–285_; Del G_–52_/A_–49_	24.20	19.23	10.9	MT725581; MT740094; MT743028
22	Sandiego	*qnrA*; *qnrB*; *qnrS*; *qepA*	*tet*(A); *tet*(B)	D87A	L121P	G186R; F148L; G112S; Ins (3 nt) T_520_; Ins (7 nt) G_319_; Ins (3 nt) C_235_; Del G_69_–A_72_	T-97C; Del T_–288_/A_–285_; Del A_–89_/T_–87_	16.85	11.77	ND	MT725582; MT725609; MT743029
23	Typhimurium	*oqxA*	*tet*(A); *tet*(B)	S83I	–	F148L; Ins (7 nt) G_319_; Ins (3 nt) C_235_; Del G_69_–A_72_	–	1.6	1.1	ND	MT725583; MT725610; MT743030
24	Magherafelt	*qnrS*; *qepA*	*tet*(A); *tet*(B)	S83A; D87Y	–	F149L; N87S; H82R; Ins (3 nt) G_493_; Ins (7 nt) G_321_; Ins (5 nt) C_252_; Del T_65_–G_69_	T-97C; V28M; Del T_–288_/A_–285_	19.25	13.22	ND	MT725584; MT725611; MT743031
25	Jedburgh	*qnrS*; *qepA*	*tet*(A); *tet*(B)	D87A	L121p	G182R; F145L; G109S; L72F; Del G_307_–C_309_; Del G_251_–C_254_; Del T_65_–G_69_	T-97C; I40L; Del T_–288_/A_–285_	14.18	10.74	ND	MT725585; MT725612; MT743032
26	Wingrove	*qnrS*; *qepA*	*tet*(A)	D87S	G181D	F147L; Ins (6 nt) C408; Del G_307_–C_309_; Del G_251_–C_254_; Del T_65_–G_69_	T-97C	6.66	4.09	ND	MT725586; MT740095; MT743033
27	Untypable	*–*	*tet*(A); *tet*(B)	D87G	L121p	F149L; A102V; N87S; H82R; Ins (7 nt) G_321_; Ins (5 nt) C_252_; Del T_65_–G_69_	T-97C; C-109T	9.44	6.42	ND	MT725587; MT725613; MT743034
28	Typhimurium	*qnrS*; *qepA*, *oqxB*	*tet*(A)	D87Y	–	F149L; N87S; H82R; Ins (7 nt) G_321_; Ins (5 nt) C_252_; Del T_65_–G_68_	T-97C	5.41	3.82	ND	MT725588; MT740096; MT743035
29	Typhimurium	*qnrA*; *qnrB*; *qnrS*; *qepA*	*tet*(A); *tet*(B)	S83L; D87G	–	F148L; Ins (7 nt) G_319_; Ins (3 nt) C_235_; Del G_69_–A_72_	T-97C; Del T_–288_/A_–285_	11.33	8.74	ND	MT725589; MT725614; MT743036

*PMQR, plasmid-mediated quinolone resistance; Ins, insertion; Del, deletion; nt, nucleotide; ND, not detected. Isolate numbers in bold were sensitive to tigecycline and resistant to ciprofloxacin. Other isolates exhibited co-resistance to ciprofloxacin and tigecycline. All isolates were nalidixic acid resistant.*

*^*a*^Mutations were detected after comparison with the respective gene in the complete genome of *S. enterica* serovar Typhimurium LT2 (GenBank accession number NC_003197).*

*^*b*^GenBank accession numbers were assigned for *gyrA*, *tet*(A), and *ramR-A* genes, respectively.*

DNA sequencing of the QRDRs of *gyrA* gene revealed specific stepwise point mutations in the topoisomerase target gene; those were associated with nalidixic acid and CIP resistance in 26 out of 29 (89.66%) XDR *Salmonella* isolates. As presented in Table 3, single mutations in *gyrA* gene either at S83 (9/26; 34.62%) or D87 (8/26; 30.77%) were detected in either low (MIC = 2 μg/ml; 5/29), moderate (MIC = 4–16; 8/29), or high (MIC = 32–128 μg/ml; 4/29) levels of CIP resistance. However, nine XDR isolates (CIP MIC = 16–64 μg/ml) were associated with double mutations at both positions. No mutation was reported in the QRDR of *gyrA* among three *Salmonella* isolates that showed nalidixic acid resistance and low-level CIP resistance (MIC = 2 μg/ml). The amino acid substitution at S83F was the most frequent mutation being detected in 23.08% of the isolates followed by D87Y (19.23%).

As depicted in Tables 2 and 3, *Salmonella* isolates (code Nos. 3, 11, and 29) contained double amino acid substitutions at S83 and D87 in QRDR of *gyrA* along with four to six PMQR determinants that had elevated MIC values for CIP (32–64 μg/ml). However, isolates of code Nos. 19 and 21 harbored single amino acid substitutions in QRDR of *gyrA* gene (S83V and D87Y, respectively) as well as six PMQR genes with CIP MICs of 4 and 128 μg/ml, respectively. Some isolates (code Nos. 15, 16, and 20) had up to three PMQR genes with no mutations in *gyrA* gene and showed a low level of CIP resistance (MIC = 2 μg/ml). On the other hand, other isolates of code Nos. 13, 23, and 27 had a single amino acid substitution, and no or just one PMQR gene showed moderate (MIC = 4–8 μg/ml) or low (MIC = 2 μg/ml) levels of CIP resistance. These results suggest that the acquisition of *gyrA* mutations as well as PMQR determinants were not the primary cause of the resistance to nalidixic acid and CIP in *S. enterica* serovars, and additional resistance mechanisms, such as enhanced efflux pump activity, may be involved.

### Impact of *tet* Genes and Mutations of the *tet*(A) Gene on TIG Resistance

The *tet*(A) and *tet*(B) genes were detected in 29 (100%) and 18 (62.07%) of the XDR *Salmonella* isolates, whereas *tet*(X4) gene was detected in only one isolate (3.45%) (code No. 18, Tables 2, 3), and *tet*(M) gene was not detected. To determine whether *tet*(A)-positive isolates had mutations in the *tet*(A) interdomain loop region, the amplicons (402 bp) from all isolates were sequenced. As shown in Tables 2, 3, comparisons of *tet*(A) gene sequences of *Salmonella* isolates with that of the wild type *Salmonella* Typhimurium reference strain (accession number NC_003197) revealed two-point mutations in the interdomain loop region of the efflux pump at codons 120 and 121 in eight TIG-resistant (MIC = 4–16 μg/ml) and one TIG-sensitive (MIC = 2 μg/ml) isolates. Moreover, a point mutation at codon 181 was observed in three isolates (MIC = 4–16 μg/ml); this important residue is located near the transmembrane domains of the efflux pump (codons 201–203) where mutations accumulated in these domains have a functional role in the efflux of TIG.

### *RamRA* Mutations Induce Efflux Genes Expressions and Confer Relevant Resistance

To investigate the impact of non-target mutations on the expression of the AcrAB-TolC efflux system and consequently on the level of CIP and TIG resistance, DNA sequencing of the *ramRA* regulatory locus of the 29 XDR *Salmonella* isolates was performed then compared with the wild-type *ramRA* gene of *Salmonella* Typhimurium reference strain (accession number NC_003197). As depicted in Table 3 and Supplementary Figure 1, screening for non-target mutations of the *ramRA* region revealed various amino acids alterations and frameshift mutations. In *ramR* gene open reading frame (ORF), the amino acid substitution *F*_145__–__150_ → L and the nucleotide deletion at G_64_-A_72_ were the predominant mutations, being detected in 28 (96.5% each) XDR isolates, followed by the insertion at the nucleotide T319–326 (62.07%). Meanwhile, deletion at T_–288_/A_–285_ was the highest detected mutation (17/29; 58.5%) in *ramR* binding region (BR). As shown in Supplementary Table 5, deletions in ORF and BR of *ramR* gene were found in 96% (24/25) and 68% (17/25) of the co-resistant XDR isolates, respectively, whereas amino acids substitutions in both regions were found in 96 and 36% of these isolates, respectively. It is interesting that the in-frame 4-bp deletion of T_–288_/A_–285_ dominated in *ramR* BR of the 17 (68%) co-resistant XDR *Salmonella* isolates displaying 2- to 32-fold and 2- to 4-fold increased resistance to CIP and TIG, respectively, than the non-mutated isolates.

### Upregulation of *ramA*, *acrB*, and *oqxB* Efflux Pump Genes in XDR *Salmonella* Isolates

To elucidate the role of enhanced efflux activity in CIP and TIG resistance, the relative expression levels of the global transcriptional regulator *ramA* and the transporter gene *acrB* of the AcrAB-TolC efflux system were assessed via qPCR. As shown in Table 3, the relative mRNA levels of *ramA* gene were maximal (exceeded 10-fold; range = 10.0–24.2, median = 12.8) in 17 out of 25 CIP/TIG co-resistant *Salmonella* isolates (68%) when compared to the pan-susceptible *Salmonella* Tamale control isolate (assigned a value of 1). Of these 17 co-resistant *Salmonella* isolates, 9 showed high expression levels for the *acrB* gene (range = 10.73–20.9; median = 11.8). None of the isolates that were uniquely resistant to CIP (*n* = 4) exhibited this overexpression in both genes. To further confirm the role of OqxAB efflux pump, we examined the relative expression levels of the *oqxB* gene in the *oqx*AB-positive isolates. Six isolates overexpressed the *oqxB* (range = 1.1–10.9; median = 4.85), that was more pronounced in four XDR isolates. Taking together the increased MIC values of CIP (2- to 32-fold) and TIG (2- to 16-fold), MAR and efflux activity indices (Table 2), and the maximal *ramA* and *acrB* expression levels for the above mentioned 17 CIP/TIG co-resistant isolates (Table 3), it is well suggested that enhanced AcrAB-TolC efflux pump activity was the most likely mechanism underlying CIP/TIG co-resistance in XDR *S*. *enterica* serovars.

### Importance of Different Genetic Markers for the Co-resistance of *Salmonella* to Ciprofloxacin and Tigecycline

**FIGURE 2:**
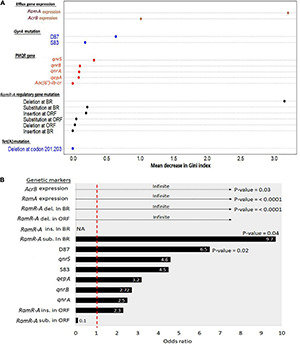
Ranked significance of genetic markers in the XDR *Salmonella* isolates that showed co-resistance to ciprofloxacin and tigecycline (*n* = 25). **(A)** Random forest classification analyses showing the importance of genetic markers in differentiating the co-resistant XDR *Salmonella* isolates from those that were resistant to CIP only (*n* = 4). The *X*-axis shows the mean decrease in Gini index for each marker. The higher the mean decrease in the Gini index, the more important the marker. **(B)** Significance of association of genetic markers and the co-resistance phenotype. The *X*-axis shows the odds ratio (at confidence intervals = 95%) of the respective genetic features that are shown on the *Y*-axis. Numbers within the bars refer to odds value. Numbers beneath the bars show the significance of association (*p*-value based on Fisher’s exact test at a cutoff level of 0.05) between the occurrence of certain genetic features and the appearance of the co-resistance to CIP and TIG in the 25 XDR isolates. The odds ratios that were shown as headed arrow indicate an infinite (∞) or maximum odds. The odds ratio of 1 (the vertical dashed red line) indicates no contribution of the respective genetic feature to the occurrence of the co-resistant phenotype. Del, deletion; Sub, substitution; Ins, insertion; BR, binding region; ORF, open reading frame.

Figure 2 shows the ranked significance of each genetic marker as a contributor to the occurrence of certain resistance patterns. The random forest classification model (Figure 2A) showed that *ramA* gene expression and deletion in *ramR* BR were the top genetic markers differentiating the CIP/TIG co-resistant isolates (*n* = 25) from those that showed resistance to CIP only (*n* = 4). The odds ratio calculation and Fisher’s exact test applied on the 25 co-resistant XDR isolates (Figure 2B) showed that the expression of *ramA* and *acrB* genes and deletion in the *ramR* BR were importantly (odds ratio = infinite) and significantly (*p* < 0.05) associated with the appearance of this phenotype.

### Associations Among Various Genetic Markers

**FIGURE 3:**
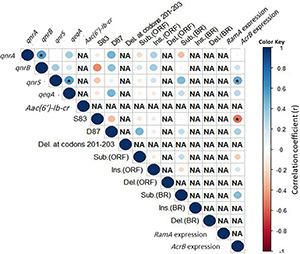
Correlation among genetic features in the co-resistant XDR *Salmonella* isolates (*n* = 25). Asterisks (*) indicate a significant correlation at a 0.05 *p*-value. The color scale refers to the correlation coefficient (blue and red colors indicate negative and positive correlations, respectively).

The correlations among pairs of the genetic markers are shown in Figure 3. In the 25 co-resistant XDR *Salmonella* isolates, certain PMQR genes were significantly positively correlated as seen in *qnrB-qnrA* and *qnrS-qepA* (*r* = 0.5 each). In the four isolates that were uniquely resistant to CIP, the highest positive correlation was found between *ramA* expression and the deletion in its BR (*r* = 1; *p* < 0.05), followed by an intermediate positive significant correlation between some members of PMQR genes. The insertion and substitution in *ramR* gene ORF were highly negatively correlated (*r* = −0.5; *p* = 0.02).

**FIGURE 4:**
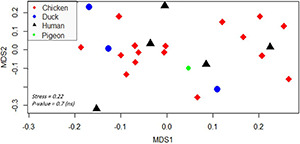
Non-metric multidimensional scaling showing the clustering and overlap of various isolates belonging to different hosts. Different hosts are color- and shape-coded. Stress refers to the performance of the multidimensional scaling. The *p*-value was calculated by PERMANOVA test and refers to the significant clustering of the groups. A *p*-value < 0.05 indicates that isolates were not significantly clustered based on their hosts (thus there is an overlap among hosts). The abbreviations of codes are described in Table 2 in detail.

Regarding the isolation source, XDR *Salmonella* isolates belonging to human and poultry hosts were largely overlapped based on the resistance phenotypes and molecular characterization of the isolates (Figure 4). This is numerically evidenced by binary distance measured between pairs of isolates (Supplementary Table 6) belonging to different hosts or based on the average distances among hosts (0.3).

## Discussion

Salmonellosis is one of the most frequent food-borne zoonoses globally, and the consumption of contaminated poultry meat is considered the main source of NTS infections in humans (Antunes et al., 2016). A tremendous increase in the antimicrobial resistance in *S*. *enterica* poses a significant global concern. While resistance to CIP in *Salmonella* isolates has been intensively studied, limited data are available on how resistance to CIP could confer resistance to other unrelated antimicrobials such as TIG, which is the last-resort anti-*Salmonella* drug. In the current study, we exploited molecular and data analysis approaches to directly investigate the molecular rationale behind the occurrence of co-resistance to CIP and TIG in XDR *Salmonella* isolates recovered from poultry and contact workers in Egypt.

The prevalence of *Salmonella* in chickens (11.54%) and pigeons (3.13%) in this study was lower than that reported in Northern China, where 20% of 1- to 4-week-old broiler chickens and 21.82% of pigeons were positive for *Salmonella*. Meanwhile, the prevalence among 1- to 4-week-old ducks (4.51%) (Wang et al., 2020) was nearly similar to that reported in our study (6.67%). The observed *Salmonella* prevalence among turkeys (4.17%) was lower than that previously reported in German fattening flocks (10.3%) (Käsbohrer et al., 2013). In contrast to the reported prevalence of *Salmonella* among quails (3.85%), a very high rate (75%) has been declared in Brazil (de Freitas Neto et al., 2013). These variations may be attributed to the geographical areas, climatic conditions, poultry species or breed, sample types, and disparity in sampling procedure and *Salmonella* isolation protocol. Serological identification of *Salmonella* isolates (*n* = 115) demonstrated that *Salmonella* Typhimurium (26.96%) and *Salmonella* Enteritidis (11.30%) were the most frequent serotypes in poultry and contact workers, which is consistent with previous reports from China (Wang et al., 2020), Egypt (Gharieb et al., 2015), and some European countries (Osimani et al., 2016).

Our data showed high resistance to amoxycillin-clavulanic acid (99.13%), cefazolin (94.78%), nalidixic acid (77.39%), cefoxitin (76.52%), tetracycline (74.78%), and cefepime (73.04%). Modest resistance rates were observed for CIP and TIG (51.30 and 24.35%, respectively). Plausible explanations of resistance are the indiscriminate use of antibiotics both in human and poultry husbandry, in addition to the easy accessibility to antibiotics in many countries worldwide, notably in Egypt. In contrast, lower resistance rates were observed for ertapenem (7.83%), imipenem (19.13%), meropenem (5.22%), and doripenem (2.61%) as there is no history of using the carbapenems for prevention or treatment in poultry farms in Egypt.

While MDR *Salmonella* represented the majority of our isolates (73.9%), we rather focused our analyses on the XDR isolates (*n* = 29; 25.22%) from poultry (*n* = 24), and contact workers (*n* = 5) because of the scarcity of data on this particular *Salmonella* phenotype in Egypt [despite its presence in other countries, e.g., *Salmonella* Typhi in Pakistan (Klemm et al., 2018; Saeed et al., 2019) and *Salmonella* Indiana in China (Wang et al., 2017)] and due to its seriousness in cases of outbreaks, as compared to MDR strains.

In the present study, we attempted to elucidate the co-resistance mechanism contributed to 25 CIP/TIG co-resistant *Salmonella* isolates. Our results revealed that the CCCP EPI was able to reverse the CIP and TIG resistance patterns for some examined *Salmonella* isolates. Therefore, we could not exclude the prospect that the AcrAB-TolC efflux pump might play a role in CIP/TIG resistance. In accordance with these findings, reduction in CIP and TIG MICs have been documented while using the CCCP EPI in previous studies (Deng et al., 2014; Zhong et al., 2014; Razavi et al., 2020). The twofold or greater decrease in the MIC levels for CIP- and TIG-resistant *Salmonella* isolates affirmed the involvement of active efflux components (Marimón et al., 2004). In a previous study, the CIP MICs decreased by twofold and those of nalidixic acid decreased by fivefold in the presence of EPIs, establishing the inclusion of an efflux pump in conversing quinolone resistance (Keddy et al., 2010). However, no complete reversion of the CIP resistance phenotype was detected with CCCP EPI in the work of Rushdy et al. (2013) implying the contribution of other mechanisms to this resistance, mainly, mutations in the target genes.

Bacterial resistance to fluoroquinolones is usually mediated by the acquisition of PMQR determinants (Robicsek et al., 2006) or mutations in bacterial DNA gyrase, particularly *gyrA* (Hopkins et al., 2005; Wasyl et al., 2014) or active efflux (Zhang et al., 2017). Herein, PMQR determinants were found in 27 XDR *Salmonella* isolates resistant to CIP (MICs ranging from 2 to 128 μg/ml), indicating the frequent occurrence of transferable quinolone resistance (Wasyl et al., 2014). This corresponds to the recent findings that all *Enterobacteriaceae* strains with high and intermediate resistance phenotypes to CIP (MICs = 1.5–512 μg/ml) harbored one or more PMQR genes (Kotb et al., 2019). This differs from the results of Szabó et al. (2018) who found that PMQR-positive *Enterobacteriaceae* strains were susceptible or intermediately resistant to CIP (MIC values between 0.06 and 1 mg/L). Notably, chromosomal copies of PMQR genes such as *oqxAB*, *aac(6′)-Ib-cr*, and *qnr*S genes have recently been discovered in CIP-resistant *Salmonella* strains (Chang et al., 2021). Thus, the presence of PMQR genes on the chromosome should not be excluded.

Previous results showed that fluoroquinolone resistance was attributed to amino acids substitutions at S83 or D87 codons within *gyrA* subunit, and these substitutions were not specific to certain *Salmonella* serovars (Robicsek et al., 2006; Chen et al., 2017). Our study revealed 16 different amino acids substitutions at both codons in 26 XDR *S. enterica* isolates representing 16 serovars, which were not associated with specific *Salmonella* serovar or CIP MIC value. Although quinolone resistance in *Salmonella* is firstly attributed to point mutations in the *gyrA* gene, other mutations of *gyrB* and *parC* genes may exist especially in higher-level resistance to fluoroquinolones. Oethinger et al. (2000) stated that the presence of *gyrA* mutations in the absence of active AcrAB efflux did not confer CIP resistance. This study’s shortcoming is not detecting *gyrB*, *parC*, and *parE* mutations; whether mutations within these genes also contribute to CIP resistance here is unknown. The distribution of *gyrA* mutations in the 25 co-resistant XDR isolates did not follow a specific pattern, and they were present in the other 4 XDR isolates showing resistance to CIP. This agrees with the results of random forest classification (i.e., having a low mean decrease in Gini index) and odds values.

Here, plasmid-encoded *tet*(X4) gene was detected in only one out of the 29 (3.45%) XDR *Salmonella* isolates (MIC = 4 μg/ml). Consistent with our results, the mobile *tet*(X) gene was recently detected in diverse pathogens resulting in TIG resistance, which is considered a public health concern (Li et al., 2020). Tuckman et al. (2000) stated that *tet*(A) mutations could induce TIG resistance in *S. enterica* isolates. Considering mutations in *tet*(A) gene, sequence analysis indicated that the point mutations of codons 120, 121, and 181 in 12 *tet(A)*-carrying isolates differed from the frameshift mutations of codons 201, 202, and 203 that reduced the sensitivity to TIG (Hentschke et al., 2010). Therefore, the contribution of these mutations to TIG resistance needs further investigation.

According to Linkevicius et al. (2016), the chemical change in position C-9 of the *tet*(B) efflux transporter gene cannot expel TIG out of the cytoplasm. In this study, the MICs of TIG for *tet*(B) positive isolates (*n* = 18) ranged from 0.5 to 16 μg/ml. TIG resistance in such *tet*(B)-carrying isolates may be attributed to *tet*(A) mutations or upregulation of *ramA*, *acrB*, and *oqxB* efflux pump genes (Table 3). A previous study indicated that the MIC for *tet*(B) positive *S. enterica* isolates (*n* = 13/49) ranged from 0.064 to 0.5 mg/L (Akiyama et al., 2013). They concluded that the mutations in *tet*(A) gene promote a low-level resistance of *S. enterica* isolates to TIG (MIC ranged from 0.19 to 3 mg/L), and additional resistance mechanisms such as *ramA* and *ramR* mutations could increase the TIG MIC to reach the resistance breakpoint.

Since the *ramRA* region have been reported to play a major role in the regulation of *ramR* and *acrB* genes expression in *Salmonella* and, consequently, in the efflux-mediated decreased susceptibility to antimicrobials (Abouzeed et al., 2008; Fàbrega et al., 2016), we investigated how mutations of *ramRA* could influence the transcription level of *ramR* and *acrB* genes and how this is linked to the occurrence of CIP/TIG co-resistance in XDR *Salmonella*. While this mechanism has been shown to play roles in *Salmonella* resistance to CIP (Fàbrega et al., 2016) or TIG (Chen et al., 2017), limited data are available on its contribution to the concurrent *Salmonella* resistance to these two drugs. A reason behind this information shortage could be the fact that TIG is a newly launched drug that has not yet received much attention. Another reason could be the lack of studies on XDR *Salmonella*, where such resistance phenotype infrequently occurs. In our analyses, deletions in *ramR* BR and *ramA* overexpression existed in 17 XDR co-resistant isolates. In addition, the random forest classification model, odds ratio, and Fisher’s exact test lend further support that these two determinants were the top two mechanisms occurring in the co-resistant XDR isolates. These data are consistent with a previous report (Fàbrega et al., 2016), although it is an *in vitro* study and involves MDR isolates. This suggests the importance of these two determinants as characteristic features of those co-resistant isolates (Figures 2A,B) and might also hint at a link between both markers as shown previously (Fàbrega et al., 2016). Coupling our data with the knowledge that *ramR* binds, through its BR, directly to the promoter of the *ramA* gene and thus controls its expression (Baucheron et al., 2012) puts forward the assumption that deletions in the *ramR* BR could impair its binding efficiency, leading to overexpression of *ramA* gene, and that the constitutive or concurrent occurrence of these two events are important for the occurrence of co-resistance to CIP and TIG in XDR *Salmonella.* This also in turn could enhance the expression of *acrB*, albeit to a low extent, and, thus, the increased MICs, MAR, and efflux activity indices. It is worth noting that although certain mutations (e.g., nucleotide deletions and amino acid substitutions in *ramR* ORF) were significantly associated with the appearance of the co-resistant phenotype and present in 96% of the 25 co-resistant isolates, respectively, their importance as determinants for such co-resistance is questionable because they also were present in the other four XDR isolates (Figure 1 and Table 3). While deletions in *ramRA* region have been linked to *acrAB* overexpression and could confer CIP (Abouzeed et al., 2008; Kehrenberg et al., 2009; Fàbrega et al., 2016) and TIG (Hentschke et al., 2010) resistance in *Salmonella*, it does not seem to be the case for the co-resistant isolates as *acrB* overexpression existed in only 36% of the co-resistant isolates.

Our analyses also pinpointed specific novel mutations at *ramR* BR in the co-resistant XDR isolates. This included deletion at T_–288_/A_–285_, which was detected in 68% of the co-resistant isolates (odds ratio = infinite and *p*-value of Fisher’s exact test ≤ 0.0001), suggesting the importance of this particular mutation in inducing such phenotype. We could not compare our results to others due to the scarcity of data on isolates exhibiting co-resistance to CIP and TIG. Similar results have also highlighted the role of *ramA* overexpression in the efflux-mediated MDR phenotype in *Salmonella* species (Fàbrega et al., 2016) and the positive correlation with the increased MICs of the antibiotics and expression of AcrAB efflux pump (Abouzeed et al., 2008). It is worthy of note that the genetic alterations reported in our study differed from those reported in the aforementioned studies and confirmed the hypothesis that sequence alterations may occur at various positions in the *ramRA* gene (Kehrenberg et al., 2009). Therefore, these mutations may have switched on the transcription of the efflux pump genes constitutively and subsequently triggered the appearance of CIP/TIG co-resistance phenotype of XDR *Salmonella*. Wong et al. (2015) reported that oqxAB, an RND efflux pump, is one of several endogenous efflux systems found in *Klebsiella pneumoniae* and *Enterobacter* species, with a role functionally comparable to that of *acrAB* in other *Enterobacteriaceae* members. A recent research reported that the oqxAB-bearing plasmid might cause *Salmonella* Typhimurium to develop a TIG-resistant phenotype. This phenomenon was probably related to the overexpression of MDR efflux pumps AcrAB-TolC and OqxAB (Chen et al., 2017). In this study, overexpression of the *oqxB* gene in four CIP/TIG co-resistant isolates indicated that the OqxAB efflux pump might be also involved in CIP and TIG co-resistance in XDR *Salmonella*. This is consistent with the findings of  Zhong et al. (2014) that AcrAB-TolC efflux pump plays an important role in TIG resistance in *K. pneumoniae* strains with MICs of 8 μg/ml, whereas both the AcrAB-TolC and OqxAB efflux pumps contributed to the TIG resistance in strains with an MIC of 16 μg/ml.

This study highlights the prevalence of *S. enterica* serovars in poultry flocks and their contact workers, particularly of the alarming XDR phenotype. The knowledge gained from this study is highly relevant in the field of antimicrobial resistance in *Salmonella* in many aspects. First, our data are deemed as an initial step in delineating the molecular rationale behind co-resistance of *Salmonella* to CIP/TIG, opening doors for the experimental validation of the proposed role of AcrAB-TolC and OqxAB efflux pumps in TIG and CIP resistance. Second, it makes it possible to understand the emergence of TIG resistance in the absence of respective selection pressure, which could be seen clinically in cases where TIG-resistant isolates emerge in *Salmonella*-infected humans or animals with no previous exposure to TIG (Hentschke et al., 2010; Chen et al., 2017).

This is the first report that concludes a variety of XDR *Salmonella* serotypes circulate in poultry flocks and their contact workers in Egypt, with *Salmonella* Typhimurium having the highest frequency. Enhanced efflux pump activity, in particular the overexpression of *ramA*, plays a fundamental role in *acrAB* overexpression and facilitates the efflux-mediated CIP/TIG co-resistance with no previous exposure to TIG. These genetic alterations suggest a potential public health concern possibly associated with the poultry-to-human transfer of resistant bacteria on farms using the antimicrobials for treatment or non-therapeutic use or both.

## Data Availability Statement

The datasets presented in this study can be found in online repositories. The names of the repository/repositories and accession number(s) can be found in the article/Supplementary Material.

## Ethics Statement

The studies involving human participants were reviewed and approved by Zagazig University Institutional Animal Care and Use Committee (ZU-IACUC) (approval number ZU-IACUC/2/F/12/2019). The patients/participants provided their written informed consent to participate in this study. The animal study was reviewed and approved by Zagazig University Institutional Animal Care and Use Committee (ZU-IACUC) (approval number ZU-IACUC/2/F/12/2019). Written informed consent was obtained from the owners for the participation of their animals in this study. Written informed consent was obtained from the individual(s) for the publication of any potentially identifiable images or data included in this article.

## Author Contributions

NA and YT contributed equally to the conception and design of the study and participated with RG in the application of classical microbiological techniques. AE carried out all PCR assays and sequencing approaches and participated in the analysis of the sequences. MS performed the bioinformatics, established the figures, and participated with MAS in statistical analyses of the data. RG, AE, MS, EK, MAS, and AA conceived the study and participated in the design. NA and YT carried out the sequence analysis and participated in the data analysis. NA, YT, and MS wrote the initial draft of the manuscript. All authors revised the manuscript critically for important intellectual content. All authors gave the final approval of the version to be published.

## Conflict of Interest

The authors declare that the research was conducted in the absence of any commercial or financial relationships that could be construed as a potential conflict of interest.

## Publisher’s Note

All claims expressed in this article are solely those of the authors and do not necessarily represent those of their affiliated organizations, or those of the publisher, the editors and the reviewers. Any product that may be evaluated in this article, or claim that may be made by its manufacturer, is not guaranteed or endorsed by the publisher.

## Footnotes

1
www.ncbi.nlm.nih.gov/BLAST/

2
https://www.expasy.org/

3
www.graphpad.com

4
https://www.r-project.org

## Supplementary Material

Supplementary Figure 1 Frequency of different mutations in the ramR-A regulatory gene as identified in the 2- investigated isolate’s group (those resistant to CIP or to both CIP and TIG).
