# Supplementary figures and images for: Extensive Drug-Resistant Salmonella enterica Isolated From Poultry and Humans: Prevalence and Molecular Determinants Behind the Co-resistance to Ciprofloxacin and Tigecycline
Source: Front Microbiol. 2021 Nov 25;12:738784. doi: 10.3389/fmicb.2021.738784 (PMC8660588; doi:10.3389/fmicb.2021.738784)

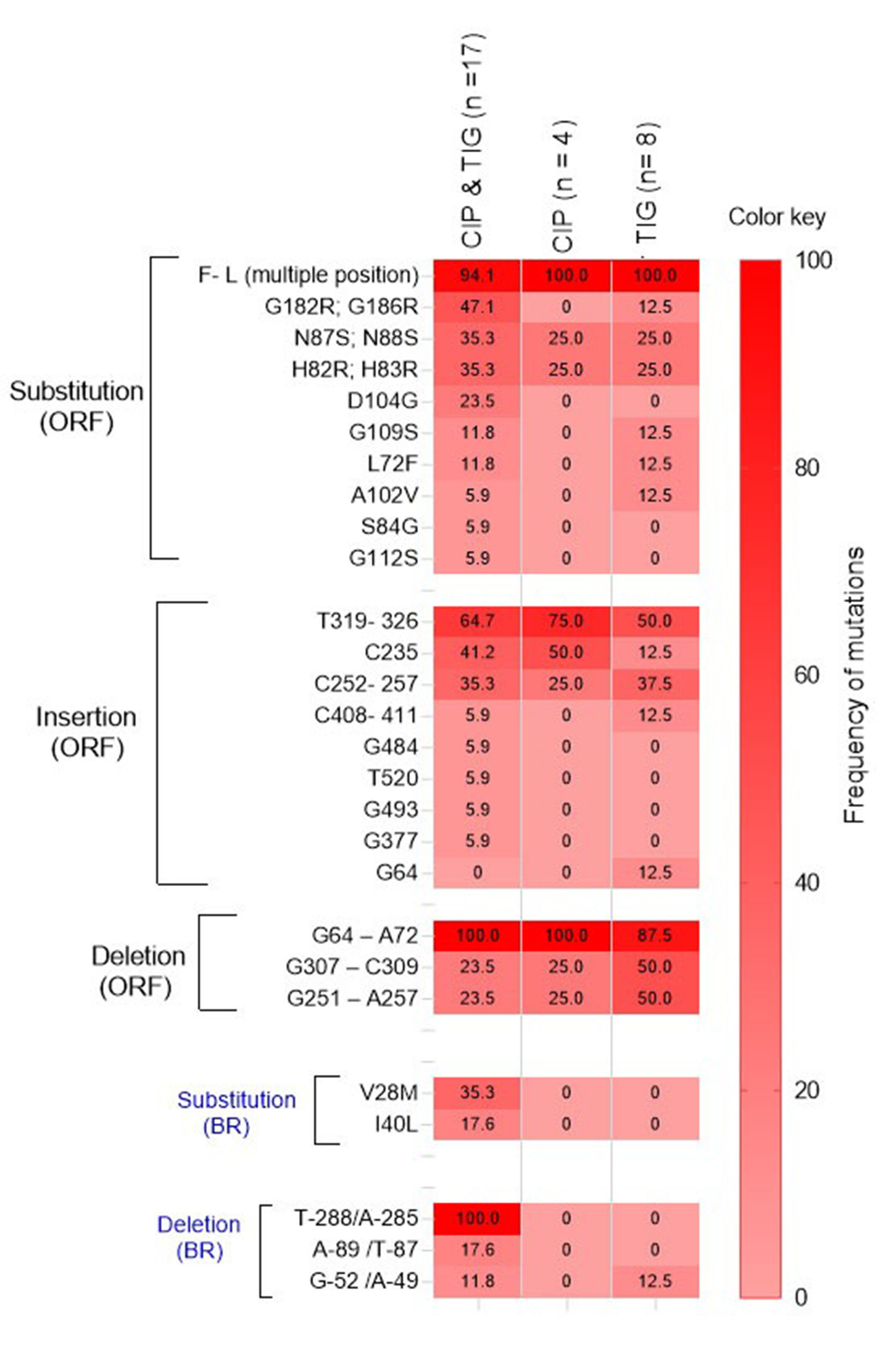

Supplement: Supplementary Figure 1 — Frequency of different mutations in the ramR-A regulatory gene as identified in the 2- investigated isolate’s group (those resistant to CIP or to both CIP and TIG). [file Image_1.jpeg]
