# Supplementary material for: Extensive Drug-Resistant Salmonella enterica Isolated From Poultry and Humans: Prevalence and Molecular Determinants Behind the Co-resistance to Ciprofloxacin and Tigecycline
Source: Front Microbiol. 2021 Nov 25;12:738784. doi: 10.3389/fmicb.2021.738784 (PMC8660588; doi:10.3389/fmicb.2021.738784)
Supplement: Supplementary file 2 [file Table_1.doc]

**Supplementary Table 1:** Oligonucleotide primer sequences used for PCR assays

| **Primer use and target gene** | **Primer name** | **Nucleotide sequence (5’→3’)** | **Annealing temperature (**˚C) | **Amplicon size (bp)** | **Reference** |
| --- | --- | --- | --- | --- | --- |
| **Salmonella identification** |  |  |  |  |  |
| *invA* | invAF  invAR | GTGAAATTATCGCCACGTTCGGGCAA  TCATCGCACCGTCAAAGGAACC | 55 | 284 | (Oliveira et al., 2003) |
| **Detection of PMQR genes** |  |  |  |  |  |
| *qnrA* | qnrAF  qnrAR | ATTTCTCACGCCAGGATTTG  GATCGGCAAAGGTTAGGTCA | 53 | 516 | (Robicsek et al., 2006) |
| *qnrB* | qnrBF  qnrBR | GATCGTGAAAGCCAGAAAGG  ACGATGCCTGGTAGTTGTCC |  | 469 |  |
| *qnrS* | qnrSF  qnrSR | ACGACATTCGTCAACTGCAA  TAAATTGGCACCCTGTAGGC |  | 417 |  |
| *qepA* | qepAF  qepAR | CGTGTTGCTGGAGTTCTTC  CTGCAGGTACTGCGTCATG | 50 | 403 | (Cattoir et al., 2008) |
| *Aac(6′)-Ib-cr* | Aac(6′)-Ib-crF  Aac(6′)-Ib-crR | CCCGCTTTCTCGTAGCA  TTAGGCATCACTGCGTCTTC | 55 | 113 | (Lunn et al., 2010) |
| **Detection of *tet* genes**  *tet(X1)* | tet(X1)-F  tet(X1)-R | CGA AAA ATG TTG CTT GGC AGC TT  AGT TGT TGA ACG AAT TAA CTC C | 59 | 486 | (Ji et al., 2020) |
| *tet(X2)* | tet(X2)-F  tet(X2)-R | CGG GAT GTC CAA GGT AAG AAA A  TGA CAA CGT CGT ATG AAT CAA |  | 343 |  |
| *tet(X3)* | tet(X3)-F  tet(X3)-R | GAC ACT TGA TCT GCA CAG GGA TT  CCC TAC AAA AGA TGA TGT CAA AC |  | 685 |  |
| *tet(X4)* | tet(X4)-F  tet(X4)-R | CTG ATT CGT GTG ACA TCA TCT TTT G  GTT AAA TTT CCC ATT GGT CAG ATT A |  | 204 |  |
| *tet(X5)* | tet(X5)-F  tet(X5)-R | GGT ATC AAC ATT TCA ATG CTT G  CGA TTC GTC CTG CGT ATC TTT TG |  | 265 |  |
| *tet*(B) | tet(B)-F  tet(B)-R | CCTTATCATGCCAGTCTTGC  ACTGCCGTTTTTTCGCC | 58 | 773 | (Van et al., 2008) |
| *tet*(M) | tet(M)-F  tet(M)-R | GTGGACAAAGGTACAACGAG  CGGTAAAGTTCGTCACACAC | 46 | 406 | (Khoshbakht et al., 2018) |
| **Detection of mutations** |  |  |  |  |  |
| *gyrA* | gyrA-F  gyrA-R | ACGTACTAGGCAATGACTGG  AGAAGTCGCCGTCGATAGAAC | 56 | 190 | (Yang et al., 2012) |
| *tet*(A) | tetA-F  tetA-R | GCCTTTCCTTTGGGTTCTCT  TGTCCGACAAGTTGCATGAT | 55 | 402 | (Li et al., 2007) |
| *ramR-ramA* | ramR1  ramR2 | CGTGTCGATAACCTGAGCGG  AAGGCAGTTCCAGCGCAAAG | 60 | 934 | (Abouzeed et al., 2008) |
| **qPCR** |  |  |  |  |  |
| *ramA* | ramA3  ramA4 | CACGATTGTCGAGTGGATTG  AAAATGCGCGTAAAGGTTTG | 58 | 232 | (Abouzeed et al., 2008) |
| *acrB* | AcrB-rt1  acrB-rt2 | GGCATTGGGTATGACTGGAC  GCATTACGGAGAACGGGATAG | 60 | 148 | (Zheng et al., 2009) |
| 16s rRNA | 16s rRNA-F  16s rRNA-R | CAGAAGAAGCACCGGCTAACTC  GCGCTTTACGCCCAGTAATT | 60 | 87 | (Botteldoorn et al., 2006) |

PMQR, plasmid mediated quinolone resistance; qPCR, quantitative PCR; bp, base pair
