# Supplementary material for: Extensive Drug-Resistant Salmonella enterica Isolated From Poultry and Humans: Prevalence and Molecular Determinants Behind the Co-resistance to Ciprofloxacin and Tigecycline
Source: Front Microbiol. 2021 Nov 25;12:738784. doi: 10.3389/fmicb.2021.738784 (PMC8660588; doi:10.3389/fmicb.2021.738784)
Supplement: Supplementary file 3 [file Table_2.doc]

**Supplementary Table 2:** Distribution of *Salmonella* serotypes among 115 isolates originated from poultry and human sources

| ***Salmonella* serotype** | **No.** | **Source (No.)** | Total (%)^a^ |
| --- | --- | --- | --- |
| Typhimurium | 22  1  1  7 | Broiler chickens [liver (8); cecum (5); muscle (5); spleen (4)]  Pigeon dropping  Turkey cecum  Human stool | 31 (26.96) |
| Enteritidis | 12  1 | Broiler chickens [cecum (4); muscle (3); liver (2); egg yolk (2); spleen (1)]  Human stool | 13 (11.30) |
| Infantis | 4  4 | Broiler chickens [liver (2); cecum (1); gall bladder (1)]  Ducks [liver (2); spleen (1); cloacal swab (1)] | 8 (6.96) |
| Kentucky | 6 | Broiler chickens [spleen (2); gall bladder (2); liver (1); cecum (1)] | 6 (5.22) |
| Newport | 4 | Broiler chicken cecum | 4 (3.48) |
| Typhi | 3 | Human stool | 3 (2.61) |
| Paratyphi C | 3 | Human stool | 3 (2.61) |
| Alfort | 3 | Pigeon [ Liver (2); dropping (1)] | 3 (2.61) |
| Tamale | 3 | Broiler chickens [cecum (2); liver (1)] | 3 (2.61) |
| Shangani | 3 | Quail [liver (2); dropping (1)] | 3 (2.61) |
| Bardo | 3 | Broiler chickens [liver (2); cecum (1)] | 3 (2.61) |
| Virchow | 1  1 | Broiler chickens cecum  Turkey cecum | 2 (1.74) |
| Magherafelt | 2 | Broiler chickens [liver (1); cecum (1)] | 2 (1.74) |
| Bargny | 2 | Broiler chickens [spleen (1); cecum (1)] | 2 (1.74) |
| Lagos | 2 | Broiler chickens [liver (1); cecum (1)] | 2 (1.74) |
| Vejle | 2 | Turkey cecum | 2 (1.74) |
| Jedburgh | 2 | Quail [liver (1); dropping (1)] | 2 (1.74) |
| Apeyeme | 1 | Turkey cecum | 1 (0.87) |
| Daula | 1 | Broiler chicken cecum | 1 (0.87) |
| Derby | 1 | Duck muscle | 1 (0.87) |
| Kiel | 1 | Broiler chicken muscle | 1 (0.87) |
| Angers | 1 | Broiler chicken cecum | 1 (0.87) |
| Molade | 1 | Broiler chicken spleen | 1 (0.87) |
| Colindale | 1 | Broiler chicken cecum | 1 (0.87) |
| Sandiego | 1 | Broiler chicken cecum | 1 (0.87) |
| Takoradi | 1 | Broiler chicken cecum | 1 (0.87) |
| Larochelle | 1 | Duck liver | 1 (0.87) |
| Labadi | 1 | Broiler chicken gall bladder | 1 (0.87) |
| Papuana | 1 | Broiler chicken gall bladder | 1 (0.87) |
| Montevideo | 1 | Broiler chicken liver | 1 (0.87) |
| Wingrove | 1 | Pigeon liver | 1 (0.87) |
| Rubislaw | 1 | Broiler chicken muscle | 1 (0.87) |
| Blegdam | 1 | Broiler chicken muscle | 1 (0.87) |
| Maloma | 1 | Broiler chicken gall bladder | 1 (0.87) |
| Anatum | 1 | Human stool | 1 (0.87) |
| Untypable | 1  4 | Broiler chicken liver  Duck [muscle (2); liver (1); cloacal swab (1)] | 5 (4.35) |

^a^ Percentages were calculated according to the total number of *Salmonella* isolates (No. = 115)
