# Supplementary material for: Extensive Drug-Resistant Salmonella enterica Isolated From Poultry and Humans: Prevalence and Molecular Determinants Behind the Co-resistance to Ciprofloxacin and Tigecycline
Source: Front Microbiol. 2021 Nov 25;12:738784. doi: 10.3389/fmicb.2021.738784 (PMC8660588; doi:10.3389/fmicb.2021.738784)
Supplement: Supplementary file 4 [file Table_3.doc]

**Supplementary Table 3: Antimicrobial resistance profiles of *Salmonella* serotypes under study**

| ***Salmonella* serotype (No)** |  | **Antimicrobial agent^a^** | | | | | | | | | | | | | | | | | | | | | | |
| --- | --- | --- | --- | --- | --- | --- | --- | --- | --- | --- | --- | --- | --- | --- | --- | --- | --- | --- | --- | --- | --- | --- | --- | --- |
|  | **AM** | **AMC** | **SAM** | **CZ** | **CXM** | **CRO** | **FEB** | **FOX** | **ETP** | **IPM** | **MEM** | **DOR** | **CN** | **TOB** | **AK** | **NA** | **CIP** | **TE** | **TIG** | **FOS** | **C** | **SXT** | **ATM** | **CT^*^** |
| **Typhimurium (31)** | **16 (51.61)** | **31 (100.00)** | **14 (45.16)** | **30 (96.77)** | **20 (64.52)** | **18 (58.06)** | **24 (77.42)** | **23 (74.19)** | **4 (12.90)** | **2 (6.45)** | **2 (6.45)** | **1 (3.23)** | **14 (45.16)** | **24 (77.42)** | **6 (19.36)** | **23 (74.19)** | **16 (51.61)** | **23 (74.19)** | **10 (32.26)** | **16 (51.61)** | **23 (74.19)** | **17 (54.84)** | **12 (38.71)** | **15 (48.39)** |
| **Enteritidis (13)** | **8 (61.54)** | **13 (100.00)** | **1 (7.69)** | **11 (84.62)** | **5 (38.46)** | **9 (69.23)** | **4 (30.77)** | **9 (69.23)** | **3 (23.08)** | **2 (15.38)** | **2 (15.38)** | **0 (0.00)** | **5 (38.46)** | **4 (30.77)** | **2 (15.38)** | **11 (84.62)** | **5 (38.46)** | **9 (69.23)** | **1**  **(7.69)** | **2 (15.38)** | **6 (46.15)** | **7 (53.85)** | **2 (15.38)** | **4 (30.77)** |
| **Infantis (8)** | **4 (50.00)** | **8 (100.00)** | **3 (37.50)** | **8 (100.00)** | **6 (75.00)** | **4 (50.00)** | **4 (50.00)** | **6 (75.00)** | **1 (12.50)** | **0**  **(0.00)** | **0 (0.00)** | **0 (0.00)** | **4 (50.00)** | **6 (75.00)** | **1 (12.50)** | **6 (75.00)** | **2 (25.00)** | **5 (62.50)** | **1 (12.50)** | **2 (25.00)** | **6 (75.00)** | **4 (50.00)** | **1 (12.50)** | **1 (12.50)** |
| **Kentucky (6)** | **4 (66.67)** | **6 (100.00)** | **3 (50.00)** | **6 (100.00)** | **5 (83.33)** | **4 (66.67)** | **4 (66.67)** | **5 (83.33)** | **1 (16.67)** | **0**  **(0.00)** | **0 (0.00)** | **0 (0.00)** | **4 (66.67)** | **4 (66.67)** | **2 (33.33)** | **5 (83.33)** | **4 (66.67)** | **5 (83.33)** | **0**  **(0.00)** | **2 (33.33)** | **3 (50.00)** | **3 (50.00)** | **4 (66.67)** | **0**  **(0.00)** |
| **Newport (4)** | **3 (75.00)** | **4 (100.00)** | **3 (75.00)** | **3 (75.00)** | **2 (50.00)** | **3 (75.00)** | **3 (75.00)** | **3 (75.00)** | **0**  **(0.00)** | **0**  **(0.00)** | **0 (0.00)** | **0 (0.00)** | **1 (25.00)** | **4 (100.00)** | **0 (0.00)** | **3 (75.00)** | **3 (75.00)** | **3 (75.00)** | **0**  **(0.00)** | **1 (25.00)** | **3 (75.00)** | **3 (75.00)** | **0**  **(0.00)** | **0**  **(0.00)** |
| **Typhi (3)** | **0 (0.00)** | **3 (100.00)** | **0**  **(0.00)** | **3 (100.00)** | **3 (100.00)** | **3 (100.00)** | **3 (100.00)** | **3 (100.00)** | **1 (33.33)** | **0**  **(0.00)** | **0 (0.00)** | **0 (0.00)** | **2 (66.67)** | **1 (33.33)** | **0 (0.00)** | **1 (33.33)** | **3 (100.00)** | **0**  **(0.00)** | **0**  **(0.00)** | **0**  **(0.00)** | **0**  **(0.00)** | **2 (66.67)** | **3 (100.00)** | **3 (100.00)** |
| **Paratyphi C (3)** | **1 (33.33)** | **3 (100.00)** | **2 (66.67)** | **3 (100.00)** | **3 (100.00)** | **3 (100.00)** | **2 (66.67)** | **3 (100.00)** | **1 (33.33)** | **0**  **(0.00)** | **0 (0.00)** | **0 (0.00)** | **2 (66.67)** | **3 (100.00)** | **2 (66.67)** | **2 (66.67)** | **1 (33.33)** | **2 (66.67)** | **1 (33.33)** | **1 (33.33)** | **1 (33.33)** | **3 (100.00)** | **3 (100.00)** | **3 (100.00)** |
| **Alfort (3)** | **1 (33.33)** | **3 (100.00)** | **1 (33.33)** | **3 (100.00)** | **2 (66.67)** | **1 (33.33)** | **2 (66.67)** | **2 (66.67)** | **1 (33.33)** | **0**  **(0.00)** | **0 (0.00)** | **0 (0.00)** | **0**  **(0.00)** | **3 (100.00)** | **0 (0.00)** | **3 (100.00)** | **1 (33.33)** | **3 (100.00)** | **1 (33.33)** | **1 (33.33)** | **2 (66.67)** | **1 (33.33)** | **1 (33.33)** | **1 (33.33)** |
| **Tamale (3)** | **1 (33.33)** | **1 (33.33)** | **1 (33.33)** | **2 (66.67)** | **1 (33.33)** | **0**  **(0.00)** | **2 (66.67)** | **0**  **(0.00)** | **0**  **(0.00)** | **0**  **(0.00)** | **0 (0.00)** | **0 (0.00)** | **1 (33.33)** | **2 (66.67)** | **0 (0.00)** | **1 (33.33)** | **0**  **(0.00)** | **1 (33.33)** | **0**  **(0.00)** | **1 (33.33)** | **2 (66.67)** | **1 (33.33)** | **0**  **(0.00)** | **0**  **(0.00)** |
| **Shangani (3)** | **1 (33.33)** | **3 (100.00)** | **1 (33.33)** | **3 (100.00)** | **1 (33.33)** | **1 (33.33)** | **3 (100.00)** | **2 (66.67)** | **0**  **(0.00)** | **0**  **(0.00)** | **0 (0.00)** | **0 (0.00)** | **1 (33.33)** | **3 (100.00)** | **0 (0.00)** | **2 (66.67)** | **2 (66.67)** | **2 (66.67)** | **0**  **(0.00)** | **1 (33.33)** | **3 (100.00)** | **2 (66.67)** | **1 (33.33)** | **1 (33.33)** |
| **Bardo (3)** | **3 (100.00)** | **3 (100.00)** | **0**  **(0.00)** | **3 (100.00)** | **2 (66.67)** | **3 (100.00)** | **2 (66.67)** | **2 (66.67)** | **2 (66.67)** | **1 (33.33)** | **1 (33.33)** | **1 (33.33)** | **1 (33.33)** | **0**  **(0.00)** | **2 (66.67)** | **3 (100.00)** | **2 (66.67)** | **3 (100.00)** | **1 (33.33)** | **1 (33.33)** | **3 (100.00)** | **3 (100.00)** | **1 (33.33)** | **1 (33.33)** |
| **Virchow (2)** | **0**  **(0.00)** | **2 (100.00)** | **0**  **(0.00)** | **1 (50.00)** | **1 (50.00)** | **0**  **(0.00)** | **2 (100.00)** | **0**  **(0.00)** | **0**  **(0.00)** | **0**  **(0.00)** | **0 (0.00)** | **0 (0.00)** | **0**  **(0.00)** | **2 (100.00)** | **0 (0.00)** | **1 (50.00)** | **0**  **(0.00)** | **1 (50.00)** | **0**  **(0.00)** | **0**  **(0.00)** | **0**  **(0.00)** | **0**  **(0.00)** | **0**  **(0.00)** | **0 (00.00)** |
| **Magherafelt (2)** | **2 (100.00)** | **2 (100.00)** | **2 (100.00)** | **2 (100.00)** | **2 (100.00)** | **2 (100.00)** | **2 (100.00)** | **2 (100.00)** | **1 (50.00)** | **1 (50.00)** | **1 (50.00)** | **1 (50.00)** | **0**  **(0.00)** | **2 (100.00)** | **0 (0.00)** | **2 (100.00)** | **2 (100.00)** | **2 (100.00)** | **2 (100.00)** | **2 (100.00)** | **2 (100.00)** | **2 (100.00)** | **0**  **(0.00)** | **2 (100.00)** |
| **Bargny (2)** | **2 (100.00)** | **2 (100.00)** | **1 (50.00)** | **2 (100.00)** | **2 (100.00)** | **2 (100.00)** | **2 (100.00)** | **2 (100.00)** | **1 (50.00)** | **0**  **(0.00)** | **0 (0.00)** | **0 (0.00)** | **1 (50.00)** | **2 (100.00)** | **0 (0.00)** | **2 (100.00)** | **1 (50.00)** | **2 (100.00)** | **0**  **(0.00)** | **0**  **(0.00)** | **2 (100.00)** | **1 (50.00)** | **1 (50.00)** | **1 (50.00)** |
| **Lagos (2)** | **2 (100.00)** | **2 (100.00)** | **1 (50.00)** | **2 (100.00)** | **1 (50.00)** | **0**  **(0.00)** | **2 (100.00)** | **1 (50.00)** | **0**  **(0.00)** | **0**  **(0.00)** | **0 (0.00)** | **0 (0.00)** | **1 (50.00)** | **2 (100.00)** | **0 (0.00)** | **2 (100.00)** | **1 (50.00)** | **2 (100.00)** | **0**  **(0.00)** | **0**  **(0.00)** | **1 (50.00)** | **1 (50.00)** | **1 (50.00)** | **0**  **(0.00)** |
| **Vejle (2)** | **1 (50.00)** | **2 (100.00)** | **1 (50.00)** | **2 (100.00)** | **2 (100.00)** | **0**  **(0.00)** | **2 (100.00)** | **2 (100.00)** | **1 (50.00)** | **0**  **(0.00)** | **0 (0.00)** | **0 (0.00)** | **0**  **(0.00)** | **2 (100.00)** | **0 (0.00)** | **1 (50.00)** | **0**  **(0.00)** | **2 (100.00)** | **0**  **(0.00)** | **0**  **(0.00)** | **1 (50.00)** | **1 (50.00)** | **0**  **(0.00)** | **0**  **(0.00)** |
| **Jedburgh (2)** | **2 (100.00)** | **2) (100.00)** | **1 (50.00)** | **2 (100.00)** | **2 (100.00)** | **2 (100.00)** | **2 (100.00)** | **2 (100.00)** | **0**  **(0.00)** | **0**  **(0.00)** | **0 (0.00)** | **0 (0.00)** | **1 (50.00)** | **2 (100.00)** | **0 (0.00)** | **2 (100.00)** | **2 (100.00)** | **2 (100.00)** | **2 (100.00)** | **2 (100.00)** | **1 (50.00)** | **2 (100.00)** | **2 (100.00)** | **2 (100.00)** |
| **Apeyeme (1)** | **1 (100.00)** | **1 (100.00)** | **1 (100.00)** | **1 (100.00)** | **1 (100.00)** | **1 (100.00)** | **1 (100.00)** | **1 (100.00)** | **0**  **(0.00)** | **0**  **(0.00)** | **0 (0.00)** | **0 (0.00)** | **0**  **(0.00)** | **0**  **(0.00)** | **0 (0.00)** | **1 (100.00)** | **0**  **(0.00)** | **1 (100.00)** | **0**  **(0.00)** | **0**  **(0.00)** | **1 (100.00)** | **0**  **(0.00)** | **0**  **(0.00)** | **0 (00.00)** |
| **Daula (1)** | **0**  **(0.00)** | **1 (100.00)** | **0**  **(0.00)** | **1 (100.00)** | **1 (100.00)** | **1 (100.00)** | **1 (100.00)** | **1 (100.00)** | **0**  **(0.00)** | **0**  **(0.00)** | **0 (0.00)** | **0 (0.00)** | **0**  **(0.00)** | **1 (100.00)** | **0 (0.00)** | **0**  **(0.00)** | **0**  **(0.00)** | **0**  **(0.00)** | **0**  **(0.00)** | **0**  **(0.00)** | **0**  **(0.00)** | **0**  **(0.00)** | **0**  **(0.00)** | **0**  **(0.00)** |
| **Derby (1)** | **0**  **(0.00)** | **1 (100.00)** | **0**  **(0.00)** | **1 (100.00)** | **0**  **(0.00)** | **1 (100.00)** | **0**  **(0.00)** | **1 (100.00)** | **1 (100.00)** | **1 (100.00)** | **0 (0.00)** | **0 (0.00)** | **0**  **(0.00)** | **1 (100.00)** | **0 (0.00)** | **0**  **(0.00)** | **0**  **(0.00)** | **1 (100.00)** | **0**  **(0.00)** | **0**  **(0.00)** | **0**  **(0.00)** | **1 (100.00)** | **0**  **(0.00)** | **1 (100.00)** |
| **Kiel (1)** | **0**  **(0.00)** | **1 (100.00)** | **0**  **(0.00)** | **1 (100.00)** | **0**  **(0.00)** | **1 (100.00)** | **0**  **(0.00)** | **1 (100.00)** | **1 (100.00)** | **1 (100.00)** | **0 (0.00)** | **0 (0.00)** | **0**  **(0.00)** | **0**  **(0.00)** | **0 (0.00)** | **0**  **(0.00)** | **0**  **(0.00)** | **1 (100.00)** | **0**  **(0.00)** | **0**  **(0.00)** | **0**  **(0.00)** | **0**  **(0.00)** | **1 (100.00)** | **1 (100.00)** |
| **Angers (1)** | **1 (100.00)** | **1 (100.00)** | **1 (100.00)** | **1 (100.00)** | **1 (100.00)** | **0**  **(0.00)** | **1 (100.00)** | **1 (100.00)** | **0**  **(0.00)** | **0**  **(0.00)** | **0 (0.00)** | **0 (0.00)** | **0**  **(0.00)** | **0**  **(0.00)** | **0 (0.00)** | **1 (100.00)** | **1 (100.00)** | **1 (100.00)** | **1 (100.00)** | **0**  **(0.00)** | **0**  **(0.00)** | **1 (100.00)** | **0**  **(0.00)** | **0**  **(0.00)** |
| **Molade (1)** | **1 (100.00)** | **1 (100.00)** | **1 (100.00)** | **1 (100.00)** | **1 (100.00)** | **1 (100.00)** | **1 (100.00)** | **1 (100.00)** | **0**  **(0.00)** | **0**  **(0.00)** | **0 (0.00)** | **0 (0.00)** | **0**  **(0.00)** | **1 (100.00)** | **0 (0.00)** | **1 (100.00)** | **1 (100.00)** | **1 (100.00)** | **0**  **(0.00)** | **0**  **(0.00)** | **1 (100.00)** | **0**  **(0.00)** | **0**  **(0.00)** | **1 (100.00)** |
| **Colindale (1)** | **1 (100.00)** | **1 (100.00)** | **1 (100.00)** | **1 (100.00)** | **1 (100.00)** | **0**  **(0.00)** | **1 (100.00)** | **1 (100.00)** | **0**  **(0.00)** | **0**  **(0.00)** | **0 (0.00)** | **0 (0.00)** | **0**  **(0.00)** | **1 (100.00)** | **0 (0.00)** | **1 (100.00)** | **1 (100.00)** | **1 (100.00)** | **0**  **(0.00)** | **0**  **(0.00)** | **1 (100.00)** | **1 (100.00)** | **0**  **(0.00)** | **0**  **(0.00)** |
| **Sandiego (1)** | **1 (100.00)** | **1 (100.00)** | **1 (100.00)** | **1 (100.00)** | **1 (100.00)** | **1 (100.00)** | **1 (100.00)** | **1 (100.00)** | **0**  **(0.00)** | **0**  **(0.00)** | **0 (0.00)** | **0 (0.00)** | **1 (100.00)** | **1 (100.00)** | **0 (0.00)** | **1 (100.00)** | **1 (100.00)** | **1 (100.00)** | **1 (100.00)** | **1 (100.00)** | **1 (100.00)** | **1 (100.00)** | **0**  **(0.00)** | **1 (100.00)** |
| **Takoradi (1)** | **1 (100.00)** | **1 (100.00)** | **1 (100.00)** | **1 (100.00)** | **1 (100.00)** | **1 (100.00)** | **1 (100.00)** | **1 (100.00)** | **0**  **(0.00)** | **0**  **(0.00)** | **0 (0.00)** | **0 (0.00)** | **1 (100.00)** | **1 (100.00)** | **0 (0.00)** | **1 (100.00)** | **1 (100.00)** | **1 (100.00)** | **1 (100.00)** | **1 (100.00)** | **1 (100.00)** | **1 (100.00)** | **0**  **(0.00)** | **1 (100.00)** |
| **Larochelle (1)** | **0**  **(0.00)** | **1 (100.00)** | **0**  **(0.00)** | **1 (100.00)** | **0**  **(0.00)** | **0**  **(0.00)** | **1 (100.00)** | **0**  **(0.00)** | **0**  **(0.00)** | **0**  **(0.00)** | **0 (0.00)** | **0 (0.00)** | **0**  **(0.00)** | **0**  **(0.00)** | **0 (0.00)** | **1 (100.00)** | **1 (100.00)** | **0**  **(0.00)** | **0**  **(0.00)** | **0**  **(0.00)** | **0**  **(0.00)** | **0**  **(0.00)** | **0**  **(0.00)** | **0**  **(0.00)** |
| **Labadi (1)** | **1 (100.00)** | **1 (100.00)** | **1 (100.00)** | **1 (100.00)** | **1 (100.00)** | **1 (100.00)** | **1 (100.00)** | **1 (100.00)** | **0**  **(0.00)** | **0**  **(0.00)** | **0 (0.00)** | **0 (0.00)** | **1 (100.00)** | **1 (100.00)** | **0 (0.00)** | **1 (100.00)** | **1 (100.00)** | **1 (100.00)** | **1 (100.00)** | **1 (100.00)** | **1 (100.00)** | **1 (100.00)** | **1 (100.00)** | **0**  **(0.00)** |
| **Papuana (1)** | **1 (100.00)** | **1 (100.00)** | **1 (100.00)** | **1 (100.00)** | **1 (100.00)** | **1 (100.00)** | **1 (100.00)** | **1 (100.00)** | **0**  **(0.00)** | **0**  **(0.00)** | **0 (0.00)** | **0 (0.00)** | **0**  **(0.00)** | **1 (100.00)** | **0 (0.00)** | **1 (100.00)** | **1 (100.00)** | **1 (100.00)** | **0**  **(0.00)** | **0**  **(0.00)** | **0**  **(0.00)** | **0**  **(0.00)** | **0**  **(0.00)** | **1 (100.00)** |
| **Montevideo (1)** | **1 (100.00)** | **1 (100.00)** | **1 (100.00)** | **1 (100.00)** | **1 (100.00)** | **1 (100.00)** | **1 (100.00)** | **1 (100.00)** | **0**  **(0.00)** | **0**  **(0.00)** | **0 (0.00)** | **0 (0.00)** | **0**  **(0.00)** | **1 (100.00)** | **0 (0.00)** | **1 (100.00)** | **0**  **(0.00)** | **1 (100.00)** | **0**  **(0.00)** | **0**  **(0.00)** | **1 (100.00)** | **0**  **(0.00)** | **0**  **(0.00)** | **1 (100.00)** |
| **Wingrove (1)** | **1 (100.00)** | **1 (100.00)** | **1 (100.00)** | **1 (100.00)** | **1 (100.00)** | **1 (100.00)** | **1 (100.00)** | **1 (100.00)** | **0**  **(0.00)** | **0**  **(0.00)** | **0 (0.00)** | **0 (0.00)** | **0**  **(0.00)** | **1 (100.00)** | **0 (0.00)** | **1 (100.00)** | **1 (100.00)** | **1 (100.00)** | **1 (100.00)** | **1 (100.00)** | **1 (100.00)** | **1 (100.00)** | **1 (100.00)** | **0**  **(0.00)** |
| **Rubislaw (1)** | **1 (100.00)** | **1 (100.00)** | **1 (100.00)** | **1 (100.00)** | **1 (100.00)** | **0**  **(0.00)** | **0**  **(0.00)** | **1 (100.00)** | **1 (100.00)** | **0**  **(0.00)** | **0 (0.00)** | **0 (0.00)** | **1 (100.00)** | **1 (100.00)** | **0 (0.00)** | **1 (100.00)** | **0**  **(0.00)** | **1 (100.00)** | **0**  **(0.00)** | **0**  **(0.00)** | **1 (100.00)** | **1 (100.00)** | **0**  **(0.00)** | **0**  **(0.00)** |
| **Blegdam (1)** | **0**  **(0.00)** | **1 (100.00)** | **0**  **(0.00)** | **1 (100.00)** | **1 (100.00)** | **1 (100.00)** | **1 (100.00)** | **1 (100.00)** | **0**  **(0.00)** | **0**  **(0.00)** | **0 (0.00)** | **0 (0.00)** | **1 (100.00)** | **0**  **(0.00)** | **0 (0.00)** | **1 (100.00)** | **0**  **(0.00)** | **1 (100.00)** | **1 (100.00)** | **1 (100.00)** | **1 (100.00)** | **1 (100.00)** | **1 (100.00)** | **1 (100.00)** |
| **Maloma (1)** | **1 (100.00)** | **1 (100.00)** | **0**  **(0.00)** | **1 (100.00)** | **1 (100.00)** | **1 (100.00)** | **0**  **(0.00)** | **1 (100.00)** | **0**  **(0.00)** | **0**  **(0.00)** | **0 (0.00)** | **0 (0.00)** | **1 (100.00)** | **0**  **(0.00)** | **0 (0.00)** | **1 (100.00)** | **0**  **(0.00)** | **1 (100.00)** | **0**  **(0.00)** | **0**  **(0.00)** | **1 (100.00)** | **1 (100.00)** | **0**  **(0.00)** | **0**  **(0.00)** |
| **Anatum (1)** | **0**  **(0.00)** | **1 (100.00)** | **0**  **(0.00)** | **1 (100.00)** | **1 (100.00)** | **1 (100.00)** | **1 (100.00)** | **1 (100.00)** | **0**  **(0.00)** | **0**  **(0.00)** | **0 (0.00)** | **0 (0.00)** | **1 (100.00)** | **0**  **(0.00)** | **0 (0.00)** | **0**  **(0.00)** | **1 (100.00)** | **0**  **(0.00)** | **0**  **(0.00)** | **0**  **(0.00)** | **1 (100.00)** | **0**  **(0.00)** | **1 (100.00)** | **1 (100.00)** |
| **Untypable (5)** | **4 (80.00)** | **5 (100.00)** | **4 (80.00)** | **5 (100.00)** | **2 (40.00)** | **1 (20.00)** | **5 (100.00)** | **4 (80.00)** | **2 (40.00)** | **1 (20.00)** | **0 (0.00)** | **0 (0.00)** | **1 (20.00)** | **4 (80.00)** | **0 (0.00)** | **5 (100.00)** | **4 (80.00)** | **4 (80.00)** | **3 (60.00)** | **3 (60.00)** | **5 (100.00)** | **5 (100.00)** | **1 (20.00)** | **3 (60.00)** |
| **Total (115)^b^** |  |  |  |  |  |  |  |  |  |  |  |  |  |  |  |  |  |  |  |  |  |  |  |  |
| **Resistant** | **67**  **(58.26)** | **114 (99.13)** | **50 (43.48)** | **109 (94.78)** | **77 (66.96)** | **70 (60.87)** | **84 (73.04)** | **88 (76.52)** | **22 (19.13)** | **9**  **(7.83)** | **6 (5.22)** | **3 (2.61)** | **46 (40.00)** | **80 (69.57)** | **15 (13.04)** | **89 (77.39)** | **59 (51.30)** | **86 (74.78)** | **28 (24.35)** | **40 (34.78)** | **76 (66.09)** | **68 (59.13)** | **38 (33.04)** | **46 (40.00)** |
| **Intermediate** | **3**  **(2.61)** | **0**  **(0.00)** | **10 (8.70)** | **4**  **(3.48)** | **32 (27.83)** | **23 (20.00)** | **23 (20.00)** | **16 (13.91)** | **12 (10.43)** | **15 (13.04)** | **6 (5.22)** | **5 (4.35)** | **6 (5.22)** | **10 (8.70)** | **12 (10.43)** | **10 (8.70)** | **41 (35.65)** | **8**  **(6.96)** | **7 (6.09)** | **14 (12.17)** | **7**  **(6.09)** | **2**  **(1.74)** | **9**  **(7.83)** | **-** |
| **Susceptible** | **45 (39.13)** | **1**  **(0.87)** | **55 (47.83)** | **2**  **(1.74)** | **6 (5.22)** | **22 (19.13)** | **8 (6.96)** | **11 (9.57)** | **81 (70.43)** | **91 (79.13)** | **103 (89.57)** | **107 (93.04)** | **63 (54.78)** | **25 (21.74)** | **88 (76.52)** | **16 (13.91)** | **15 (13.04)** | **21 (18.26)** | **80 (69.57)** | **61 (53.04)** | **32 (27.83)** | **45 (39.13)** | **68 (59.13)** | **69 (60.00)** |
| **MAR index** | **0.02** | **0.04** | **0.02** | **0.04** | **0.03** | **0.03** | **0.03** | **0.03** | **0.01** | **0.003** | **0.002** | **0.001** | **0.02** | **0.03** | **0.01** | **0.03** | **0.02** | **0.03** | **0.01** | **0.01** | **0.03** | **0.02** | **0.01** | **0.016** |

AM, ampicillin; AMC, amoxycillin-clavulanic acid; SAM, ampicillin-sulbactam; CZ, cefazolin; CXM, cefuroxime; CRO, ceftriaxone; FEB, cefepime; FOX, cefoxitin; ETP, ertapenem; IPM, imipenem; MEM, meropenem; DOR, doripenem; CN, gentamicin; TOB, tobramycin; AK, amikacin; NA, nalidixic acid;CIP, ciprofloxacin; TE, tetracycline; TIG, tigecycline; FOS, fosfomycin; C, chloramphenicol; SXT, sulfamethoxazole-trimethoprim; ATM, aztreonam; CT, colistin; MAR, multiple antibiotic resistance

*Susceptibility of *Salmonella* isolates to colistin (CT) was determined using the broth microdilution method.

Values represent number of *Salmonella* isolates (%)

^a^Percentage of resistance for each antimicrobial agent was calculated according to the number of each *Salmonella* serotype

^b^ Percentages of total resistant, intermediate or susceptible isolates to each antimicrobial was calculated based on the total number of *Salmonella* isolates (No= 115).
