# Supplementary material for: Extensive Drug-Resistant Salmonella enterica Isolated From Poultry and Humans: Prevalence and Molecular Determinants Behind the Co-resistance to Ciprofloxacin and Tigecycline
Source: Front Microbiol. 2021 Nov 25;12:738784. doi: 10.3389/fmicb.2021.738784 (PMC8660588; doi:10.3389/fmicb.2021.738784)
Supplement: Supplementary file 5 [file Table_4.doc]

**Supplementary Table 4:** [**VITEK 2**](http://www.biomerieux-diagnostics.com/vitek-2) **MIC results of antimicrobial agents against *Salmonella* serotypes under study**

| **Isolate No.** | **Serovar** | **MIC of Antimicrobial agents (μg/mL)** | | | | | | | | | | | | | | | | |
| --- | --- | --- | --- | --- | --- | --- | --- | --- | --- | --- | --- | --- | --- | --- | --- | --- | --- | --- |
|  |  | AM | SAM | CZ | CRO | FEB | ETP | IPM | MEM | TOB | AK | CN | CIP | MXF | TIG* | F | SXT | ATM |
| 1 | Typhimurium | 32 | 32 | 64 | 8 | 32 | 0.5 | 4 | 0.25 | 16 | 8 | 2 | 16 | 8 | 4 | 128 | 512 | 16 |
| 2 | Typhimurium | 2 | 128 | 32 | 16 | 16 | 0.25 | 0.25 | 1 | 32 | 16 | 16 | 32 | 4 | 4 | 128 | 128 | 32 |
| 3 | Untypable | 64 | 32 | 32 | 64 | 32 | 0.5 | 16 | 0.5 | 64 | 4 | 1 | 64 | 8 | 16 | 512 | 512 | 16 |
| 4 | Magherafelt | 32 | 64 | 128 | 8 | 64 | 0.25 | 1 | 0.25 | 16 | 4 | 4 | 16 | 4 | 4 | 128 | 256 | 2 |
| 5 | Typhimurium | 32 | 32 | 64 | 8 | 32 | 0.5 | 0.25 | 0.5 | 64 | 2 | 16 | 4 | 2 | 2 | 128 | 128 | 32 |
| 6 | Takoradi | 128 | 64 | 64 | 16 | 128 | 0.5 | 1 | 0.25 | 16 | 16 | 32 | 32 | 8 | 8 | 128 | 128 | 2 |
| 7 | Labadi | 32 | 128 | 32 | 64 | 32 | 0.25 | 0.25 | 1 | 128 | 8 | 128 | 64 | 8 | 16 | 512 | 128 | 16 |
| 8 | Typhimurium | 64 | 32 | 64 | 8 | 64 | 0.25 | 0.5 | 0.25 | 4 | 64 | 64 | 2 | 1 | 4 | 128 | 256 | 64 |
| 9 | Jedburgh | 32 | 32 | 32 | 64 | 32 | 0.25 | 4 | 1 | 16 | 16 | 4 | 64 | 8 | 8 | 512 | 512 | 16 |
| 10 | Alfort | 32 | 64 | 32 | 16 | 32 | 0.5 | 16 | 0.25 | 128 | 1 | 2 | 64 | 4 | 16 | 512 | 128 | 32 |
| 11 | Typhimurium | 128 | 4 | 128 | 8 | 256 | 0.5 | 0.25 | 0.5 | 32 | 4 | 64 | 32 | 8 | 8 | 128 | 256 | 16 |
| 12 | Typhimurium | 32 | 2 | 64 | 8 | 32 | 0.25 | 16 | 0.25 | 16 | 2 | 4 | 16 | 8 | 16 | 128 | 512 | 64 |
| 13 | Untypable | 64 | 32 | 32 | 1 | 64 | 2 | 4 | 0.5 | 2 | 16 | 2 | 8 | 1 | 4 | 128 | 512 | 16 |
| 14 | Blegdam | 2 | 4 | 64 | 16 | 32 | 0.5 | 2 | 1 | 4 | 8 | 32 | 2 | 0.25 | 4 | 256 | 256 | 16 |
| 15 | Infantis | 2 | 2 | 64 | 8 | 2 | 0.5 | 4 | 1 | 16 | 2 | 16 | 2 | 2 | 4 | 128 | 256 | 4 |
| 16 | Enteritidis | 2 | 2 | 32 | 16 | 32 | 0.25 | 0.25 | 0.25 | 1 | 64 | 1 | 2 | 1 | 4 | 128 | 128 | 32 |
| 17 | Typhimurium | 32 | 4 | 256 | 8 | 128 | 0.25 | 1 | 0.5 | 4 | 128 | 4 | 16 | 8 | 8 | 512 | 256 | 16 |
| 18 | Paratyphi C | 64 | 32 | 64 | 16 | 32 | 0.5 | 0.25 | 0.25 | 2 | 256 | 64 | 2 | 2 | 4 | 128 | 256 | 64 |
| 19 | Typhimurium | 32 | 2 | 128 | 8 | 64 | 0.5 | 0.5 | 0.25 | 4 | 64 | 16 | 4 | 4 | 0.5 | 256 | 512 | 16 |
| 20 | Typhimurium | 2 | 32 | 64 | 1 | 32 | 0.5 | 1 | 1 | 16 | 16 | 32 | 2 | 1 | 4 | 128 | 128 | 16 |
| 21 | Bardo | 32 | 4 | 32 | 64 | 128 | 4 | 16 | 16 | 4 | 64 | 4 | 128 | 8 | 32 | 512 | 128 | 32 |
| 22 | Sandiego | 128 | 32 | 64 | 16 | 32 | 0.5 | 0.25 | 0.25 | 32 | 4 | 16 | 32 | 8 | 16 | 128 | 256 | 16 |
| 23 | Typhimurium | 32 | 2 | 128 | 16 | 64 | 0.5 | 2 | 1 | 4 | 128 | 64 | 4 | 2 | 0.5 | 128 | 512 | 64 |
| 24 | Magherafelt | 64 | 64 | 32 | 64 | 32 | 16 | 16 | 8 | 16 | 2 | 1 | 32 | 8 | 16 | 512 | 256 | 4 |
| 25 | Jedburgh | 32 | 2 | 64 | 8 | 32 | 0.5 | 0.25 | 0.25 | 128 | 8 | 128 | 64 | 8 | 16 | 128 | 256 | 32 |
| 26 | Wingrove | 32 | 32 | 32 | 8 | 256 | 0.25 | 1 | 0.25 | 16 | 16 | 4 | 2 | 1 | 4 | 128 | 512 | 16 |
| 27 | Untypable | 2 | 64 | 64 | 1 | 32 | 0.5 | 0.25 | 0.5 | 64 | 1 | 32 | 2 | 2 | 4 | 512 | 128 | 1 |
| 28 | Typhimurium | 64 | 32 | 64 | 16 | 1 | 8 | 4 | 4 | 16 | 16 | 64 | 8 | 2 | 1 | 128 | 128 | 4 |
| 29 | Typhimurium | 2 | 128 | 32 | 8 | 2 | 0.5 | 0.25 | 0.25 | 32 | 4 | 16 | 32 | 8 | 8 | 128 | 256 | 1 |

AM, ampicillin; SAM, ampicillin-sulbactam; CZ , cefazolin; CRO, ceftriaxone, FEB, cefepime; IPM, imipenem; ETP, ertapenem; MEM, meropenem; TOB, tobramycin; AK, amikacin; CN, gentamicin; CIP, ciprofloxacin; MXF, Moxifloxacin; TIG, tigecycline; F, Nitrofurantoin; SXT, sulfamethoxazole-trimethoprim; ATM, aztreonam.

The MICs were determined by [VITEK® 2](http://www.biomerieux-diagnostics.com/vitek-2) (bioMérieux, Marcy L'Étoile, France) testing using AST-GN91 cards (SKU Number: 414780; <https://www.yumpu.com/it/document/read/38357654/gn-ast-terminating-2013>). MICs for TIG were performed and interpreted according to CLSI guidelines.
