# Supplementary material for: Extensive Drug-Resistant Salmonella enterica Isolated From Poultry and Humans: Prevalence and Molecular Determinants Behind the Co-resistance to Ciprofloxacin and Tigecycline
Source: Front Microbiol. 2021 Nov 25;12:738784. doi: 10.3389/fmicb.2021.738784 (PMC8660588; doi:10.3389/fmicb.2021.738784)
Supplement: Supplementary file 6 [file Table_5.doc]

**Supplementary Table 5:** Genetic determinants contributed to CIP and TIG resistance in XDR *Salmonella* isolates

| **Feature** | | **CIP resistant***  **(n = 4)** | **CIP/TIG resistant**  **(n = 25)** | **Total**  **(n = 29)** |
| --- | --- | --- | --- | --- |
| Plasmid mediated quinolone resistance (PMQR) | *qnrA* | 2 | 14 | 16 |
|  | *qnrB* | 1 | 7 | 8 |
|  | *qnrS* | 2 | 18 | 20 |
|  | *qepA* | 3 | 23 | 26 |
|  | *oqxA* | 3 | 3 | 6 |
|  | *oqxB* | 3 | 4 | 7 |
|  | *oqxAB* | 2 | 4 | 6 |
|  | *Aac(6′)-Ib-cr* | 0 | 0 | 0 |
| *tet* genes | *tet*(A) | 4 | 25 | 29 |
|  | *tet*(B) | 1 | 17 | 18 |
|  | *tet*(M) | 0 | 0 | 0 |
|  | *tet*(X) | 0 | 1 | 1 |
| *gyrA* mutation | S83 | 3 | 15 | 18 |
|  | D87 | 1 | 16 | 17 |
| *tetA* mutation | del. at codons 201-203 | 0 | 0 | 0 |
| *ramR-A* regulatory gene mutations | *ramR* ORF (Substitution) | 4 | 24 | 28 |
|  | *ramR* ORF (Insertion) | 3 | 19 | 22 |
|  | *ramR* ORF (Deletion) | 4 | 24 | 28 |
|  | *ramR* binding region (Substitution) | 0 | 9 | 9 |
|  | *ramR* binding region (Insertion) | 0 | 0 | 0 |
|  | *ramR* binding region (Deletion) | 0 | 17 | 17 |
| Efflux genes expression level  (Those that are ˃ 10 fold) | *RamA* | 0 | 17 | 17 |
|  | *AcrB* | 0 | 9 | 9 |

CIP, ciprofloxacin; TIG, tigecycline; del, deletion; ORF, open reading frame

*The number of isolates in CIP and CIP/TIG phenotype refers to the uniquely resistant isolates
